# The Role of the Electronic Structure during Protein Folding through Electron Density-Based Quantum Chemical Descriptors

**DOI:** 10.1021/acsomega.5c05968

**Published:** 2026-01-10

**Authors:** Acassio Rocha-Santos, Igor Barden Grillo, Gabriel Aires Urquiza-Carvalho, Gerd Bruno Rocha

**Affiliations:** † Department of Chemistry, 28097 Federal University of Paraíba, Cidade Universitária, João Pessoa, Paraíba 58051-900, Brazil; ‡ Department of Chemistry, 28116 Federal University of Pernambuco, Cidade Universitária, Recife, Pernambuco 50670-901, Brazil

## Abstract

One of the major challenges in protein folding is understanding the role that the electronic structure of proteins plays during their folding. We emphasize that the structural and dynamic properties of proteins are extremely important for understanding how their conformational changes occur during folding. However, since the electronic structure is intrinsically related to the atomic structure, further analysis of the electronic structure during folding may assist in the development of methods for predicting protein biological activity. In this study, we applied statistical sampling in molecular dynamics folding trajectories, and subsequent calculations of global and local quantum chemical molecular descriptors calculated by DFT-D3 and semiempirical quantum chemical methods for three fast-folding proteins (NTL9, BBA, and α3D). We observe an intriguing trend in the local hardness per residue (η_*j*_). Specifically, soft residues do not become softer as the trajectory progresses until they reach the expected softness, and hard residues do not become progressively harder. Rather, a subtle process occurs in which the local hardness fluctuates above and below the final native values for each residue. The point is not that the folded structures have more favorable hard or soft interactions in their residues, but that η_*j*_ becomes stable as the conformation approaches the folded state. In addition, we observed that η_*j*_ can distinguish non-native from native-like structures, revealing that intrinsic aspects of the electronic structure play a highly relevant role in the protein folding process. These observations could show an electronic structure signature during protein folding.

## Graphical abstract

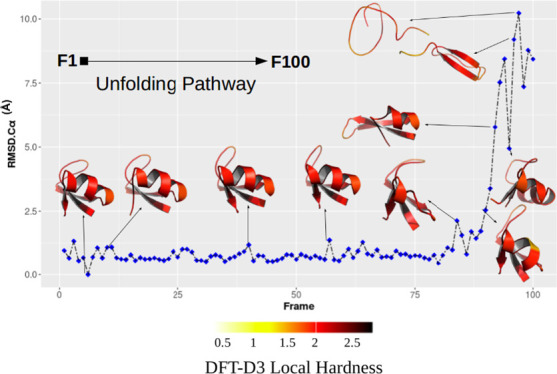

## 1 Introduction

The problem of protein folding has eluded researchers for nearly six decades. Although deceptively simple to illustrate, this challenge remains one of the main open problems at the interface between chemistry, physics, computer science, mathematics, and biology.^1–7^

Several experimental techniques and theoretical strategies have been applied to the study of the protein folding problem. Among the experimental techniques, we can list X-ray crystallography,^8^ NMR spectroscopy,^8^ laser temperature jumps,^9,10^ near-edge X-ray absorption fine structure (NEXAFS) spectroscopy,^11,12^ circular dichroism,^10,13,14^ infrared spectroscopy^15–18^ and fluorescence measurements.^10^ Theoretical strategies include molecular dynamics (MD) simulations^19–24^ as well as enhanced sampling methods,^25,26^ such as replica exchange molecular dynamics (REMD),^27–31^ umbrella sampling,^32,33^ and metadynamics.^34–37^

Because protein folding takes place on time scales ranging from milliseconds to seconds, performing full-atom unfolding/folding molecular dynamics (MD) simulations^19^ requires high-performance computers with thousands of processors and GPU-type accelerators and terabytes worth of disk storage, even for the simplest proteins. Despite these setbacks, many researchers have applied MD simulations to study the protein folding process, revealing subtle but important aspects.^9,10,23,24,29,38–51^

These MD simulations have become frequent since the appearance of modern force fields, such as Amber (Amber99SB-ILDN and Amber99SB*-ILDN) and CHARMM (CHARM27, CHARMM22*, and CHARMM36).^19,52^ However, even considering such advances, these force fields still fail to calculate thermodynamic properties, such as folding enthalpy and thermal capacity.^52^ Moreover, it is challenging to accurately model unfolded or disordered proteins and to predict the propensity of each amino acid to form specific secondary structures.^53^

The native structure of a protein is typically stabilized by a wide variety of interatomic interactions, such as (i) hydrogen bonds, (ii) van der Waals interactions, (iii) electrostatic interactions, and (iv) hydrophobic interactions.^1,54^ Considering that such interactions arise from electronic effects, such as atomic polarizability, dipole–dipole, and molecular orbital overlap, the electronic structure is required for the complete study of protein folding.^55,56^ However, understanding the proper role of the electronic structure in the protein folding process is a very difficult theoretical-computational challenge to overcome. However, despite this difficulty, some work has made advances toward a solution.^45,57–65^

Recently, Culka et al. published three interesting studies in which they addressed the problem of protein folding through the combined use of MD and quantum chemical methods.^66–68^ They have textually stated that “It is understood, and it has also been shown in this work, that the protein folding problem is too complex to be described by simple quantities such as the interaction and strain energies as presented here”.^68^ This captures the overall experience of trying to make sense of the mountains of data generated by these types of computation. The sheer volume of data to process is daunting, but the difficulties extend beyond this consideration alone. The electronic properties of protein folding are also difficult to determine because the process itself is so nuanced that no one is sure which features are essential to the process and which are variations of the core model.

Another study worth mentioning was performed by Ianeselli et al.^45^ The authors showed that molecular dynamics simulations of folding pathways combined with quantum chemical calculations of the circular dichroism spectrum can complement the circular dichroism technique applied in the folding process, since this provides time-dependent information that is impossible to capture experimentally. They exemplified this for the folding of canine milk lysozyme proteins with excellent results.

In principle, any quantity calculated using quantum chemistry methods, whether or not an observable property, can be classified as a quantum chemical molecular descriptor (QCMD).^69^ The list is rather long. In addition to the molecular quantities themselves, it is possible to propose transformations or compositions of currently known descriptors to generate new ones. Examples of descriptors include atomic charges, thermochemical quantities, electronic energies, energies of molecular orbitals, dipole moments, ionization potentials, locations of frontier molecular orbitals, electron density in atoms, and so on.

Currently, QCMDs based on electron density have aroused the interest of researchers in Quantitative Structure Activity­(Property) Reactivity (QSAR/QSPR) studies.^70,71^ This is a consequence of the fact that the electron density is fundamentally related to the molecular Hamiltonian and, therefore, the source of all molecular properties, both in the ground state and in the excited states.^72^ Another important point is that the electron density can be calculated or obtained experimentally by applying several techniques.

However, given the present computational power and the use of efficient methods and algorithms that have enabled the modeling of molecular systems with many atoms, the challenge is now to calculate QCMDs based on the electron density for biomolecules, where it is very common to find structures that exceed thousands of atoms. Thus, our group developed PRIMoRDiA^73–76^ (https://github.com/igorChem/PRIMoRDiA1.0v), an open-source tool intended to calculate many QCMDs for large systems. The use of software tailor-made for large systems is necessary to address the volume of data produced by a study such as this one.

A predictor of chemical reactivity known for its excellent results when applied to small molecules is the hard/soft acid–base principle (HSAB).^61^ It is possible to transform this predictor into a well-defined quantum descriptor by computation through Fukui functions, a reactivity descriptor calculated based on the conceptual density functional theory, CDFT.^77^

Using PRIMoRDiA, it is possible to make sense of the mountains of data produced by quantum chemical methods when they are applied to biomolecules. In fact, software has been used to tackle enzymatic catalysis, producing results that could guide the proposal or refinement of catalysis mechanisms.^78^ Furthermore, our group has shown, in another study, that Fukui functions can be calculated by PRIMoRDiA from semiempirical electron structures with an accuracy equivalent to that of their ab initio and DFT counterparts, such as those obtained from the B3LYP DFT calculations.^79^ This indicates that by using PRIMoRDiA, it is possible to achieve simultaneous accuracy and speed, a rare privilege when problems of theoretical and computational chemistry are approached.

Because PRIMoRDiA makes it possible to efficiently calculate QCMDs for biological systems as well as MD folding trajectories, we can evaluate the role of the electronic structure in such an elaborate process. In short, in this study, we calculated several QCMDs for structures along the protein folding/unfolding paths for BBA^80^ (PDB-ID: 1FME), NTL9 ^81^ (PDB-ID: 2HBA), and α3D^82^ (PDB-ID: 2A3D), which were produced by Lindorff-Larsen et al.^42^ These calculations allowed us to evaluate aspects of the electronic structure during their folding/unfolding processes.

## 2 Methods

In this study, we explore the reaching native state (RNS)/leaving native state (LNS) trajectories of the proteins NTL9, BBA, and α 3D to understand the role the electronic structure plays during their folding processes through the calculation of QCMDs. The trajectories for these small proteins were retrieved from http://www.deshawresearch.com/index.html (access to these trajectories is restricted and may be granted upon formal request to the original authors).^42^

This study was carried out following a series of computational procedures and analyses shown in Figure 1. We summarized the steps as follows: (i) sampling of the raw data to obtain time-uncorrelated structures; (ii) for each structure in the sample, single-point quantum chemical calculations (B3LYP-D3 and PM7 semiempirical method) were performed to produce electronic structures to be analyzed by PRIMoRDiA software; (iii) calculation of quantum chemical descriptors for all sampled structures; (iv) statistical analysis for all structures in the sample.

**Figure 1:**
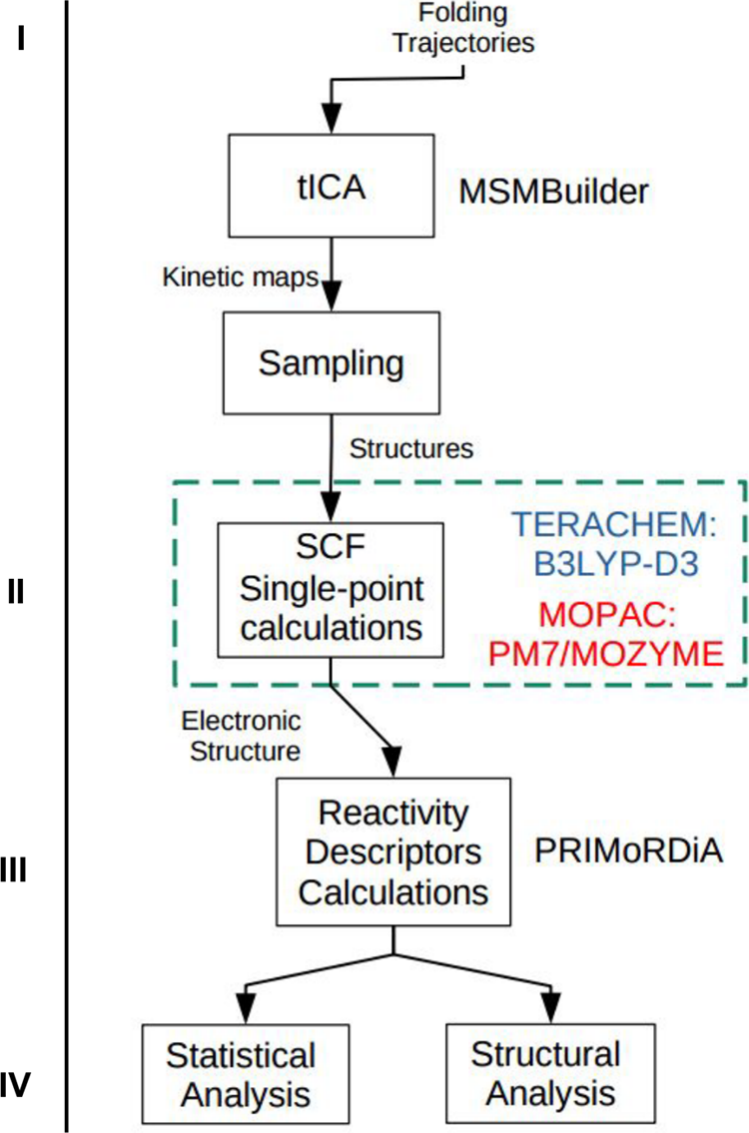
Flowchart showing the information pipeline and the computational procedures performed in this study.

### 2.1 Time-Lagged Independent Component Analysis

Time-Lagged Independent Component Analysis (tICA) is a dimensionality reduction method that uses linear transformations to identify the slowest modes of motion in time series, such as molecular dynamics trajectories, allowing the differentiation of metastable conformational states and the analysis of kinetic transitions important for understanding the mechanism of protein folding.^83^

First, we separately aligned the structures of NTL9, BBA, and α3D along their RNS/LNS trajectories,^42^ using the MDtraj program.^84^ The trajectories are 377, 223, and 346 μs long, respectively. These simulation times are compatible with the experimental data for this set of fast-folding proteins.^42^ Then, we analyze MD trajectories using the time-structure-based independent component analysis (tICA) method^85–87^ with the MSMbuilder program.^88^

For this, we chose the dihedral angles ψ and φ of the protein backbones as features and applied tICA to reduce the dimensionality of the systems. The kinetic maps of the first two tICA coordinates were plotted to evaluate the free energy of the surface. In this section, we constructed kinetic maps using the protocol available on the website of the MSMbuilder program,^88^ http://msmbuilder.org/3.8.0/tutorial.html, the chosen lag time values being on the same order of magnitude as the lag time values from previous studies applied to these proteins.^89,90^ The lag times used for the tICA analyses were 10 ns, 0.5 μs, and 10 ns for the NTL9, BBA, and α3D proteins, respectively. The optimal lag time depends on the rate at which a protein explores its conformational space and undergoes transitions between metastable states, making it a system-specific parameter.^83^ As we observed that the *x* axes served as good folding or unfolding coordinates for the NTL9, BBA, and α3D proteins (Figure 2), we sampled 100 conformations for each protein along these axes for future quantum chemical treatment.

**Figure 2:**
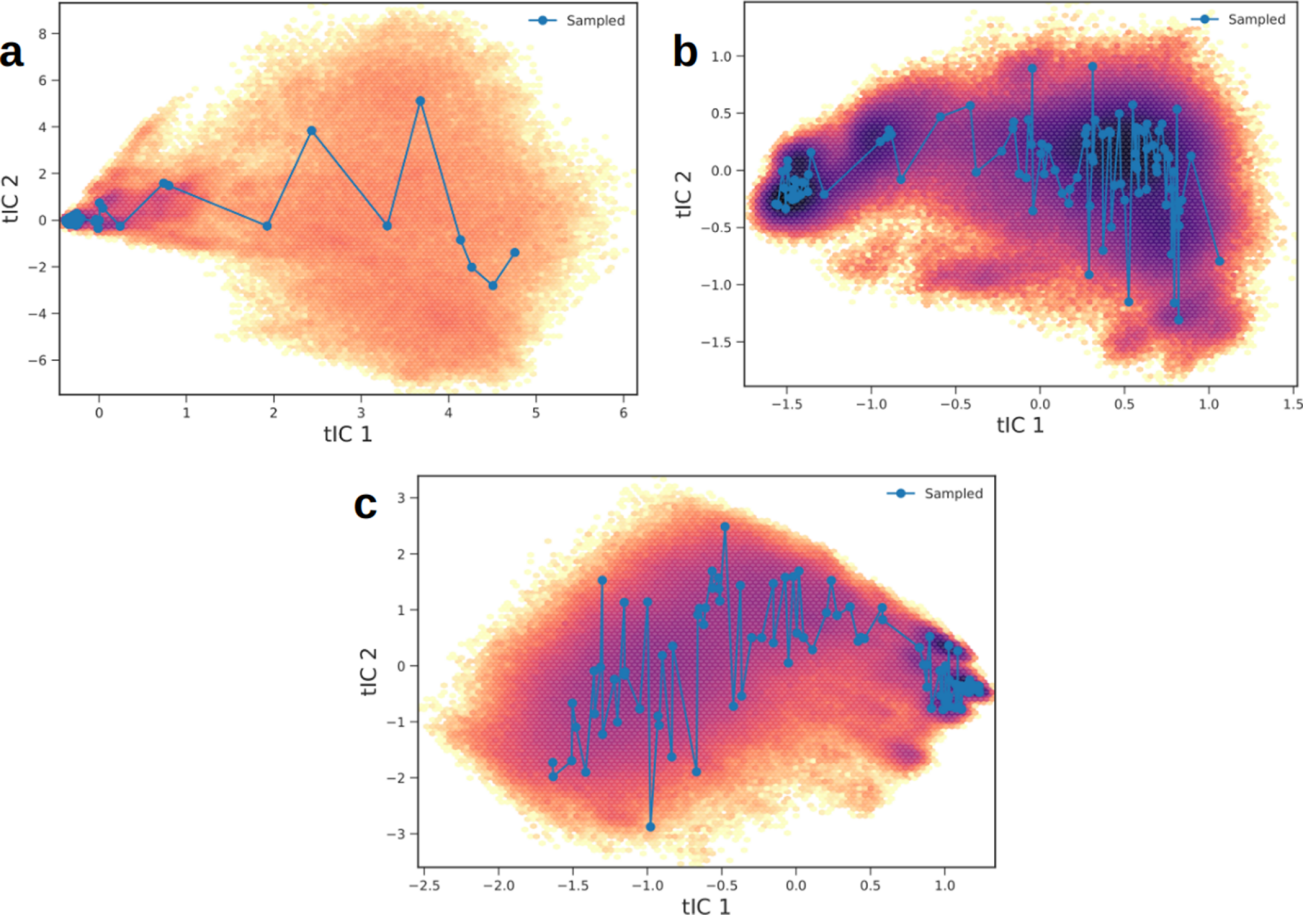
Kinetic map of the first two coordinates of time-independent components (tIC1 and tIC2) for the (a) NTL9, (b) BBA, and (c) α3D proteins. The more intense the purple color, the lower the energy.

### 2.2 Quantum Chemical Calculations

We performed single-point energy calculations for tICA sampled structures using TeraChem^91,92^ with the hybrid GGA B3LYP functional,^93,94^ considering D3 ^95^ dispersion correction and the PCM^96^ solvation model (EPS = 78.4). In addition, we also performed the same calculations using the semiempirical quantum chemistry method^97^ by using the MOPAC2016 program.^98,99^ For semiempirical calculations, we used the MOZYME^100^ linear scaling algorithm and the implicit solvation model of COSMO (EPS = 78.4 and RSOL = 1.3). Thus, we performed 300 single-point calculations via DFT and 300 single-point calculations via PM7, totaling 600 quantum chemical calculations.

From these single-point energy calculations, we calculated two sets of QCMDs: global QCMDs and local ones per residue. We also used *E*_ele_ from the TeraChem output for the B3LYP-D3 calculations. From the electron density, other global QCMDs were calculated for the PM7 and B3LYP-D3 methods using PRIMoRDiA.^73,74^

Using the RNS/LNS coordinates, we also calculated the fraction of native contacts (*Q*) and the RMSD-C_α_ of all conformations, taking the crystallographic structure as a reference. Finally, we performed DSSP, RMSF, and RMSD calculations per amino acid residue for comparison with results from local reactivity descriptors. All additional results are shown in the Supporting Information material.

### 2.3 Quantum Chemical Molecular Descriptors

The theoretical framework of QCMDs is the Conceptual-DFT, which binds well-established chemical concepts to constants retrieved from the fundamental differential equation of the DFT.^77^ This equation, shown in eq 1, describes the change in electronic energy d*E* with respect to the number of electrons d*N* and the electronic density ρ­(*r*), considering an external potential dν­(*r*), all functionals of position *r*.

dE=μdN+∫ρ(r)dν(r)dr 1

The electronic chemical potential (ECP) is the first global descriptor derived in CDFT eq 1, symbolized by the Greek letter μ, and it is the Lagrange multiplier in the problem of energy minimization considering the constant external potential.^101^ This means that this quantity represents how much the energy will change when there is a variation in the number of electrons. The name comes from the analogous thermodynamic property, chemical potential, due to the same ability to indicate the achievement of equilibrium, but in electron transfer processes.

The ECP can be estimated by the electronic energy of the frontier molecular orbitals, the highest occupied molecular orbital (HOMO), and the lowest unoccupied molecular orbital (LUMO), considering the frozen orbital approximation (FOA). We can consider such quantities as quantum chemical descriptors as well as the ionization potential (IP) and electron affinity (EA). In Table 1, we show the definition in the CDFT of the ECP and other global descriptors in the FOA approach.

**Table 1:** Global Reactivity Descriptors Computed via PRIMoRDiA

global descriptor	CDFT definition	calculation method	ref
ionization potential (IP)		–*E*_HOMO_	
electron affinity (EA)		–*E*_LUMO_	
chemical potential (μ)	${\left(\frac{\partial E}{\partial N}\right)}_{\nu }$	$\frac{E_{\mathrm{HOMO}}+E_{\mathrm{LUMO}}}{2}$	^102^
hardness (η)	${\left(\frac{\partial^{2}E}{\partial N^{2}}\right)}_{\nu }$	*E*_LUMO_ – *E*_HOMO_	^101^
softness (*S*)	1/η		^101^
electrophilicity (ω)	$\frac{\mu^{2}S}{2}$		^103^
*n*_Max_	$-\frac{\omega }{\eta }$		^103^

The second derivative of the electronic energy with respect to the number of electrons, or the derivative of the ECP with respect to the number of electrons, is the resistance to changing the number of electrons in the system, which was directly connected with the chemical hardness proposed by Pearson,^104^ where its reciprocal is defined as the chemical softness. As Klopman determined, the source of the activation energy, or the driving forces, in chemical reactions is of two types:^105^ controlled by charges and controlled by orbitals, which are the hard–hard and soft–soft interactions, respectively. The total electrophilicity ω is another global descriptor and is calculated to estimate the total loss of electronic energy when the system receives electrons from an ideal donor. And *N*_max_ is the maximum number of electrons that the system can receive, considering its total electrophilicity and hardness.

The local QCMDs can be obtained in the atom-condensed representation, that is, where values of local quantities are assigned individually to each atom with the nucleus coordinate as the reference position. In PRIMoRDiA^73^ software, we also have a condensed-residue representation, where the values assigned to the atoms are summed for each amino acid residue, which improves the interpretation of biological polymers and makes statistical models easier to understand.

The first local descriptor obtained from eq 1 is the change in electron density with respect to the number of electrons, as shown in eq 2.^106^ This descriptor was named after Fukui due to his work on the role of frontier molecular orbitals in reactivity, and it describes the regions where the electron density will move to make the new bonds and from where it will be removed.^107^

$$f(r)={\left(\frac{\partial \rho (r)}{\partial N}\right)}_{\nu }$$ 2

Thus, one of the most powerful functions in explaining/predicting reactivity is divided into two other functions: the left Fukui function (eq 3), which describes the sites susceptible to electrophilic attacks; and the right Fukui function­(eq 4), which indicates the sites prone to nucleophilic attacks. This division occurred due to the finite differences method in resolving the derivative, using simple variations of one plus/less electron, because such particles are integers and can not render a continuous function. Although such a division gave us two distinct functions that were also used to generate the Fukui radical susceptibility function and a dual Fukui function, the former being the average, as shown in eq 5 of left and right, and the latter being the subtraction of the right by the left demonstrated in eq 6, giving net behavior at point *r*.

$$f^{-}(r)=\rho (r{)}_{N}-\rho (r{)}_{N-1}$$ 3

$$f^{+}(r)=\rho (r{)}_{N+1}-\rho (r{)}_{N}$$ 4

$$f^{0}(r)=\pm \frac{f^{-}(r)+f^{+}(r)}{2}$$ 5

$$f^{\pm }(r)=f^{+}(r)-f^{-}(r)$$ 6

The FOA method simplifies the calculation of these descriptors, making the left Fukui function equal to the density of HOMO and the right equal to the density of LUMO.^108^ In addition to the success of the application of Fukui functions with FOA for small molecules, their use for energy-degenerate systems is limited due to the contribution of multiple molecular orbitals near (in energy) HOMO and LUMO.^76^ Molecules with one or more aromatic rings or biological copolymers, such as proteins and DNA-like structures, suffered from a lack of account in the other molecular orbitals before the work of our group in developing and applying modified Fukui functions for these cases.^73,78^ One of these methods, Energy Weighted (EW), is the one used in this study, where the remaining Fukui functions are defined in eq 7, as the weighted sum of the densities of the occupied molecular orbitals with the maximum energy difference from HOMO that defines the set *b*. The same repeats for the right Fukui function, but is changed by virtual molecular orbitals, considering LUMO as a reference.

$$f_{\mathrm{EW}}^{-}=\sum_{i=b}^{\mathrm{HOMO}}e^{-|E_{i}-E_{\mathrm{HOMO}}|}|\psi_{i}{|}^{2}$$ 7

$$f_{\mathrm{EW}}^{+}=\sum_{i=\mathrm{LUMO}}^{b}e^{-|E_{i}-E_{\mathrm{LUMO}}|}|\psi_{i}{|}^{2}$$ 8

Fukui functions are required in other local QCMDs, such as local softness, which is just a distribution of the global quantity when the number of electrons is replaced by the electron density, as shown in eq 9. This has a direct influence on the interpretation of Fukui function, being a local predictor of intramolecular soft–soft interactions, because of the representation of the changing capacity of the electron density, i.e., it is not expected that hard species are locally well represented by Fukui functions because of the high potential of variability of the electron density; therefore, it is not expected in the hard–hard interaction site.

$$s(r)={\left(\frac{\partial \rho (r)}{\partial \mu }\right)}_{\nu }={\left(\frac{\partial N}{\partial \mu }\right)}_{\nu }{\left(\frac{\partial \rho (r)}{\partial N}\right)}_{\nu }=Sf(r)$$ 9

Thus, for well-characterized hard–hard reactions, a definition of local hardness is required. However, by analyzing the result of the reciprocal of eq 9, given in eq 10, we cannot derive an analytical solution because of the lack of a local version of the chemical potential, and when we apply the chain rule to this derivative, it yields only an expression that does not integrate with the global quantity. An alternative strategy bypasses the integration constraint by calculating the derivative directly from the DFT functionals (eq 11), resulting in three definitions of local hardness. A key derivation employs the Thomas–Fermi–Dirac (TFD) model. In regions far from nuclei, the kinetic and exchange-correlation contributions from the TFD model become negligible, simplifying the expression to one dominated by the electron–electron interaction term.^109^ This simplification produces a practical working equation (eq 12) that is consistent with the CDFT and highlights the role of electrostatics.

$$\eta (r)={\left(\frac{\partial \mu }{\partial \rho (r)}\right)}_{\nu }$$ 10

$$\eta (r)={\left(\frac{\partial \mu }{\partial \rho (r)}\right)}_{\nu }=\frac{1}{2N}\int \frac{\delta^{2}F[\rho ]}{\delta \rho (r)\delta \rho (r^{\prime })}\rho (r^{\prime })dr^{\prime }$$ 11

$$\eta^{\mathrm{TFD}}(r)=\frac{1}{2N}\int \frac{\rho (r^{\prime })}{\left|r-r^{\prime }\right|}dr^{\prime }$$ 12

Within this framework, a further approximation substitutes the total electron density with the left Fukui function, *f* ^–^(**r**), which represents the reactive regions of the density. Omitting the 1/2*N* normalization factor then simplifies the equation to the form known as the Fukui potential η­(**r**) (eq 13).^110^ This potential measures the force exerted on the nuclei by variations in electron number, thereby capturing the change in local hardness at specific atomic sites. The approach can be generalized by using the right (*f* ^+^) or zero (*f* ^0^) Fukui functions to derive alternative variants.

$$\eta (r)=\int \frac{f^{-}(r^{\prime })}{\left|r-r^{\prime }\right|}dr^{\prime }$$ 13

A third approach formulates the local hardness problem by defining a local chemical potential (LCP) through manipulations of functional derivatives.^111^ This LCP-based definition inherently satisfies the global integration constraint. The result, given in eq 14, expresses local hardness as a function that combines the global chemical potential (μ), global hardness (η), and position-dependent electron density terms.

$$\eta (r)=\left(\rho (\mathrm{HOMO})-\frac{\rho (r)}{N}\right)\frac{\mu }{2N}+\frac{\rho (r)}{N}\eta$$ 14

A final and more intuitive method defines local hardness by distributing the global value using the Fukui function as a weight^112^ (eq 15). This model is based on the premise that hard electrophiles have a high ionization potential (IP) and low electron affinity (EA), favoring electrostatic control, while hard nucleophiles also maximize IP and minimize EA. However, this approach has been criticized for its dependence on the Fukui function, which creates conceptual redundancy with softness analyses and offers limited new insight into hard–hard interaction profiles, a limitation corroborated by our own findings:^78^

$$\eta (r)=\mathrm{IP}\cdot f^{-}(r)-\mathrm{EA}\cdot f^{+}(r)$$ 15

The local hardness calculated from the electron–electron interaction of the molecular electrostatic potential equation is the most successful in finding patterns in the folding trajectories among the various local hardness alternatives available in the PRIMoRDiA software.^73,113^ It is important to mention that this definition of local hardness does not integrate into the global quantity and is not the direct inverse of local softness.

## 3 Results and Discussion

### 3.1 Kinetic Maps

The kinetic maps for the first two coordinates of time-independent components (tIC1 and tIC2) are shown in Figure 2 for the (a) NTL9, (b) BBA, and (c) α3D small proteins. Analyzing these data, we can observe that the conformations for the folded states for both NTL9 and BBA are on the left in Figure 2a,b, respectively, and on the right side in Figure 2c for α3D. Therefore, the *x*-axis (tIC1) corresponds to the unfolding coordinates for both NTL9 and BBA and the folding coordinate for α3D. Based on this result, we sampled 100 conformations along the tIC1 coordinate for the three small proteins studied in this work to perform quantum chemical calculations.

By evaluating the coordinates of tIC1 from right to left, we observed that the NTL9 protein quickly reached its native state. However, for the BBA protein, a completely different behavior was observed. We can see that the free energy surface of the BBA protein is much rougher, with several local minima along the *x*-axis. As a natural consequence, the BBA protein takes a long time to reach its native state. The α3D protein has an intermediate roughness compared to the NTL9 and BBA proteins, as determined by examining the coordinates of tIC1 from left to right. These data corroborate the observations obtained by Beauchamp et al.^89^ and Dickson and Brooks,^114^ which demonstrated that the BBA protein exhibits multistate behavior; therefore, its folding cannot be described by a two-state model, in contrast to NTL9 and α3D, which showed homogeneous behavior and thus their folding mechanisms can be accurately described by a two-state model.

### 3.2 Assessing Global QCMDs along the RNS/LNS Path

Our next step was to calculate the global reactivity descriptors (using B3LYP-D3 and PM7) along the trajectories obtained by the first two tICA coordinates for the proteins NTL9, BBA, and α3D. We analyze the correlation between all QCMDs and the structural properties of RMSD-C_α_ and *Q* using the pairwise correlation map depicted in Figures S1–S3 (PM7 calculations) and Figures S7–S9 (B3LYP-D3 calculations). By analyzing our data, we can determine that the values of *R*, the correlation coefficient, between all global QCMDs and structural properties, RMSD-C_α_ and *Q*, are meaningless for all proteins considering both applied quantum chemical methods. In fact, such global electronic properties alone tend not to be related with common studied biological processes, such as protein–ligand and enzymatic catalysis.^76^ This happens because the ionization potential or frontier molecular orbital energies for copolymers may not be significant because of several sites where electrons could be abstracted/absorbed. The proteins studied here are small in comparison with those relevant for biological phenomena, even though we believe that they do not contain useful information that can be associated with the studied process. More complete versions of these graphs are shown in Figures S4–S6 for PM7 and Figures S10–S12 for B3LYP-D3.

Semiempirical Δ*H*_f_ and *E*_ele_ were the only quantities that showed moderate correlations with RMSD-C_α_ and *Q*, presenting |*R*|s values ranging between 0.57 and 0.86 and 0.46–0.79, respectively. The semiempirical results corroborate findings from the study of Urquiza-Carvalho et al.,^62^ which concluded that Δ*H*_f_ in aqueous solution is a good scoring function to discriminate native structures in a set of decoys.

This behavior may be expected, as these small proteins are not active enzymes, which is consistent with the results obtained by Milosavljevic et al.,^11^ who showed that the electronic core-to-valence shell transition energies are not considerably impacted by protein unfolding. The authors suggested that the frontier orbitals remain strongly localized.

### 3.3 Assessing Local QCMDs along the Folding/Unfolding Path

Our main discussion of the role of the electronic structure in protein folding is focused on the local QCMDs that were obtained for all residues along the trajectories. As the results obtained through DFT and PM7 were similar, the entire discussion of the results is based on DFT calculations, and the PM7 results are presented in the Supporting Information with similar conclusions.

We generated heatmaps for all local QCMDs listed in the Methods section for all 100 structures of each protein and compared them with heatmaps of the structural properties per residue: RMSF, RMSD, and DSSP.

The heatmaps show that the local QCMDs EAS, NAS, RAS, frontier molecular orbitals, and electrophilicity did not produce relevant patterns along the folding/unfolding trajectories of any of the three proteins studied when considering both quantum chemical methods. However, the local hardness (η_*j*_, where *j* refers to a specific residue) and the local electron density (ρ_*j*_, where *j* refers to a specific residue) presented interesting behavior, which is correlated with structural properties such as RMSD, RMSF, and DSSP. The heatmaps obtained with local hardness and local electron density data showed the same pattern; however, the local hardness data render better visualization because this feature is more sensitive to small variations of amino acid residues. Thus, our discussion is based on the local hardness. The results for the local electron density can be seen in Figures S18 and S19 (B3LYP-D3) and Figures S27 and S28 (PM7).

We can observe in Figure 3a that there are regions evincing higher values of hardness than others, more specifically, α-helix and β-sheet regions. However, this effect is not homogeneous, as there are fewer hard residues in the secondary structures. We also found that variations occur in the harder regions of the protein during unfolding.

**Figure 3:**
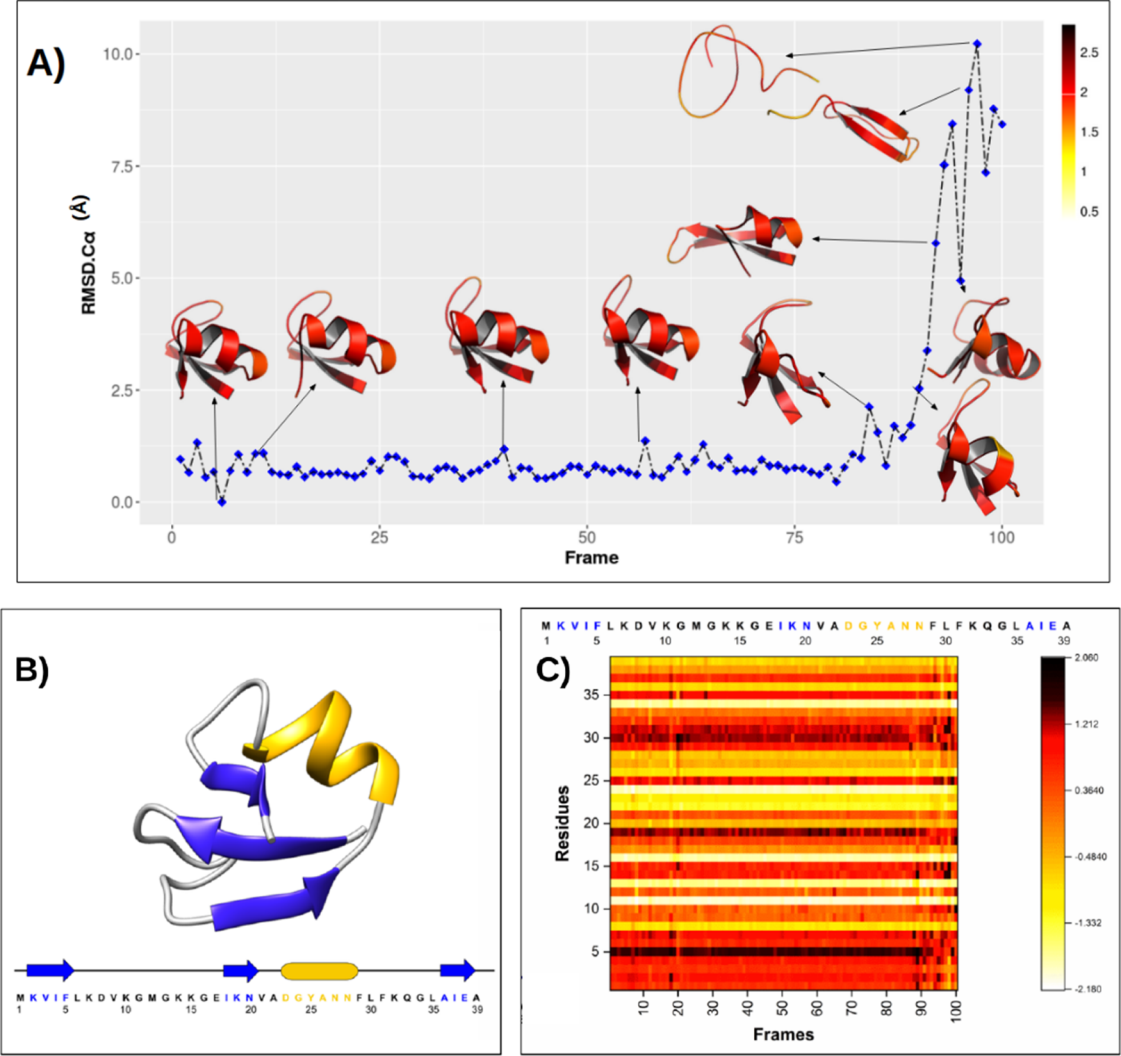
(A) Representations of 10 conformations along the trajectory obtained from the *x* coordinate of tICA for unfolding of NTL9. RMSD-Cα are shown on the *y* axis. Local hardness is represented as a color palette applied to the depicted backbone in which black represents harder regions and white represents softer regions. (B) Structural representation of the native state of the NTL9 protein (PDB-ID: 2HBA). Shown in blue are the β-sheets, and in gold, the α-helices. In addition, the sequence of the primary structure of the NTL9 protein is presented, where the amino acids belonging to the secondary structures are colored. (C) Local hardness heatmap for the 100 conformations of the NTL9 folding path. The local hardness scales in figure A and C are different because in figure C the data was normalized and centered at the origin to compare the heatmaps of the three proteins evaluated.

When analyzing Figure 3C, we observe that the local hardness pattern changes as the RMSD-C_α_ decreases. When the NTL9 protein reaches RMSD-C_α_ ≤ 2.5 Å (frame 90), the local hardness assumes more stable values; that is, the softness and hardness of the residues stop fluctuating. In this way, we can distinguish between non-native structures (frames 100-91) and native-like structures (frames 90-1), revealing a much more detailed view of the free energy surface.

From this finding, it is possible to predict whether the structure descends the free energy funnel toward the native structure by monitoring the local hardness patterns per residue. The stabilization behavior of local hardness was observed to follow the same pattern observed in RMSD-C_α_ (Figure S13a), RMSF (Figure S14a), and DSSP (Figure S15). From right to left (folding), RMSD-C_α_ and RMSF decrease rapidly, reaching stable values per residue from frame 90 to frame 1, the segment of the trajectory where the local hardness stops fluctuating.

The formation of α-helix and β-sheets secondary structures seems to follow the same pattern; that is, the formation of all secondary structures highlighted in Figure 3B begins at frame 90, causing the protein NTL9 to assume its native state by the time it reaches frame 1.

Unlike what we observed for the NTL9 protein, the free energy surface of the BBA protein is rougher, with the protein visiting more intermediate metastable states along the folding pathway. Similarly to that observed in NTL9, we verified that the local hardness is more concentrated in the secondary structures; however, the residues in the α-helices seem to have greater local hardness than the residues in the β-sheets.

The heatmap of Figure 4C together with secondary structure data (Figure S16) shows that there is greater stability of local hardness from frame 25 to frame 1, where the α-helix is partially formed (residues: 15–19) and one β-sheet is formed (residues: 10–12). At this point, the RMSD-C_α_ reduces to a local minimum (frame 20), where we observe the complete formation of the α-helix (residues: 15–24) and the formation of two β-sheets (residues: 3–4 and 11–12).

**Figure 4:**
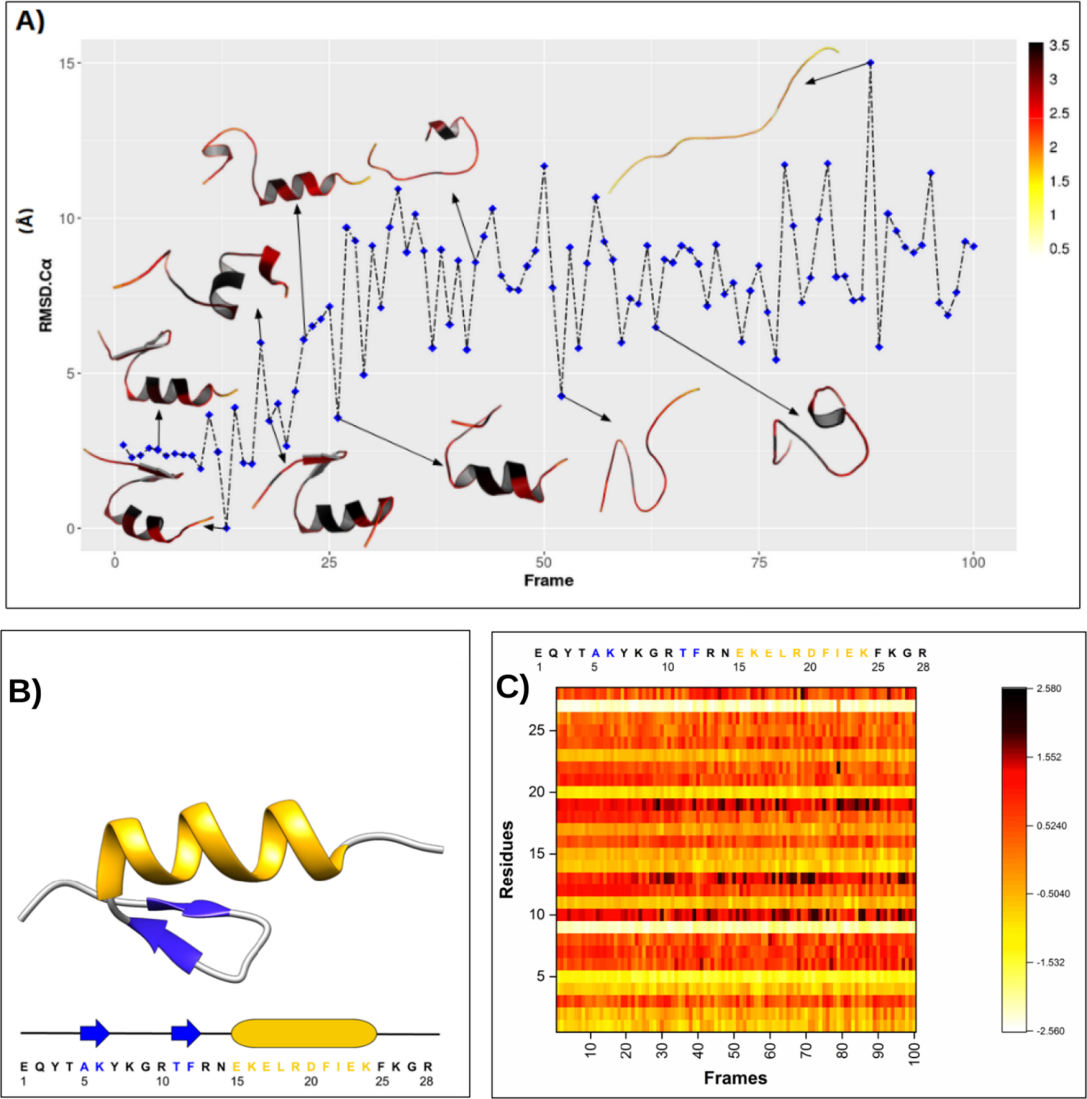
(A) Representations of 10 conformations along the trajectory obtained from the *x* coordinate of tICA for unfolding of the BBA. RMSD-Cα are shown on the *y* axis. Local hardness is represented as a color palette applied to the depicted backbone, in which black represents harder regions and white represents softer regions. (B) Structural representation of the native state of the BBA protein (PDB-ID: 1FME). Shown in blue are the β-sheets, and in gold, the α-helices. In addition, the sequence of the primary structure of the BBA protein is presented, where the amino acids belonging to the secondary structures are colored. (C) Local hardness heatmap for the 100 conformations of the BBA unfolding path. The local hardness scales in figure A and C are different because in figure C the data was normalized and centered at the origin to compare the heatmaps of the three proteins evaluated.

From frame 19, one of the β-sheets in the BBA protein migrates from residues 3–4 to residues 5–7, showing similarity to the position of the crystallographic structure (residues: 5–6). In addition, we observed that the RMSD-Cα increases from frame 19, where the two β-sheets (residues: 5–7 and 10–12) are formed, and the α-helix is partially formed (residues: 15–21). RMSD-Cα decreases again from frame 16, when there is an increase in the number of α-helix residues (15–23), reaching the lowest energy state in frame 13, when the number of α-helix residues is maximized (15–25), and the local hardness remains stable until the end of the trajectory (frame 1). In this way, the local hardness regions can be divided into two parts: (1) non-native structures (frames 100-26) and (2) native-like structures (frames 25-1) determined by the change in the local hardness pattern of Figure 4C.

In Figure 5A, we find a trend similar to that observed for the NTL9 and BBA proteins; that is, although the global hardness does not vary much along the trajectory, we observe that the local hardness changes considerably along the folding direction. We can see that the locality of full or partially formed secondary structures has a higher local hardness, but there is no homogeneity, as there are unfolded regions with a high local hardness.

**Figure 5:**
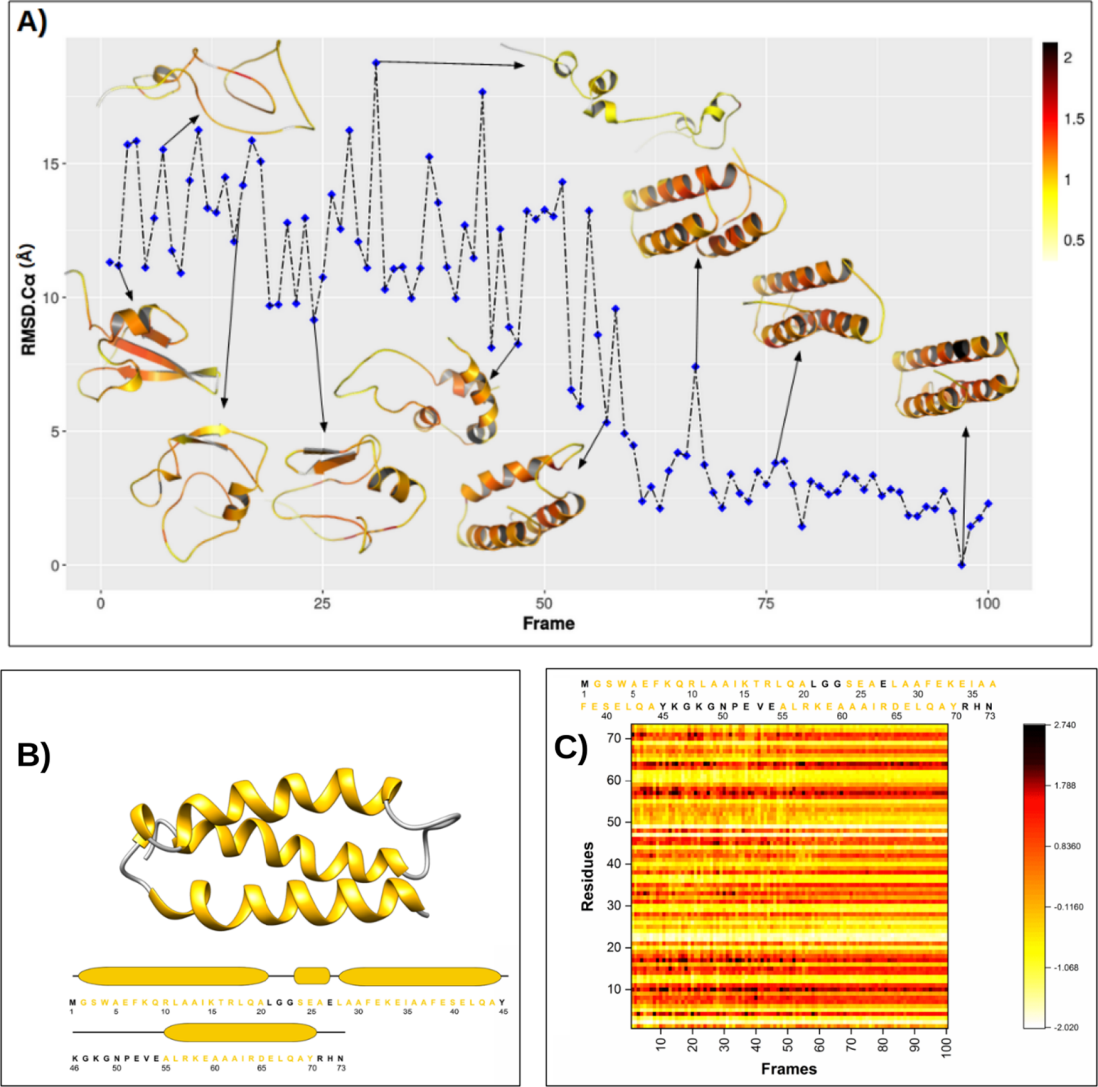
(A) Representations of 10 conformations along the trajectory obtained from the *x* coordinate of tICA for the folding of the α3D. RMSD-Cα are shown on the *y* axis. Local hardness is represented as a color palette applied to the depicted backbone in which black represents harder regions and white represents softer regions; (B) structural representation of the native state of α3D protein (PDB-ID: 2A3D). The secondary structures of α-helices are represented in gold. In addition, the sequence of the primary structure of the α3D protein is presented, where the amino acids belonging to secondary structures are colored. (C) Local hardness heatmap for the 100 conformations of the α3D folding path. The local hardness scales in figure A and C are different because in figure C, the data are normalized and centered at the origin to compare the heatmaps of the three proteins evaluated.

Similar to that observed for the NTL9 and BBA proteins, we observed that as the α3D structure approaches the native structure, the local hardness values become more stable. We noticed that the non-native structures start from the beginning of the folding path (frame 1) and extend to frame 58. From frame 59, we notice a change in the local hardness patterns, where the values stabilize, becoming relatively constant.

Therefore, we can separate the data from Figure 5C into two regions: non-native structures (frames 1–58) and the region where the local hardness stabilizes, called native-like structures (frames 59–100). We found that these data corroborate what was verified in the heatmaps for RMSD-Cα (Figure S13c) and RMSF ([Fig fig5]) per residue, where they assume relatively constant values starting from frame 59. The similarity to the DSSP analysis (Figure S17) is also clear, where the formation of the three α-helices occurs exactly from frame 59, reaching the native state conformation. These data reveal that for α3D protein folding, it is first necessary to form all secondary structures, suggesting a diffusion-collision mechanism.

The aforementioned local hardness patterns along the trajectory become much more evident when heatmaps were generated with the local Δη_*j*_ for the three proteins studied. The Δη_*j*_ for each residue during the RNS/LNS trajectory was calculated as Δη_*j*_^*i*^ = η_*j*_^*i*^ – η_*j*_^*r*^, where the subscript *j* runs over all residues in a protein, and the superscript *i* runs over all frames along the trajectory. The frame with the lowest RMSD-Cα in the trajectory was denoted with the superscript *r* (the reference frame for the calculation of Δη_*j*_^*i*^). Therefore, the reference frames for the three proteins studied were: 6 (NTL9), 13 (BBA), and 97 (α3D).

A positive value for Δη_*j*_^*i*^ indicates that residue *j* in frame *i* is harder than the same residue in the folded reference structure. The results for the three proteins are summarized in Figure 6.

**Figure 6:**
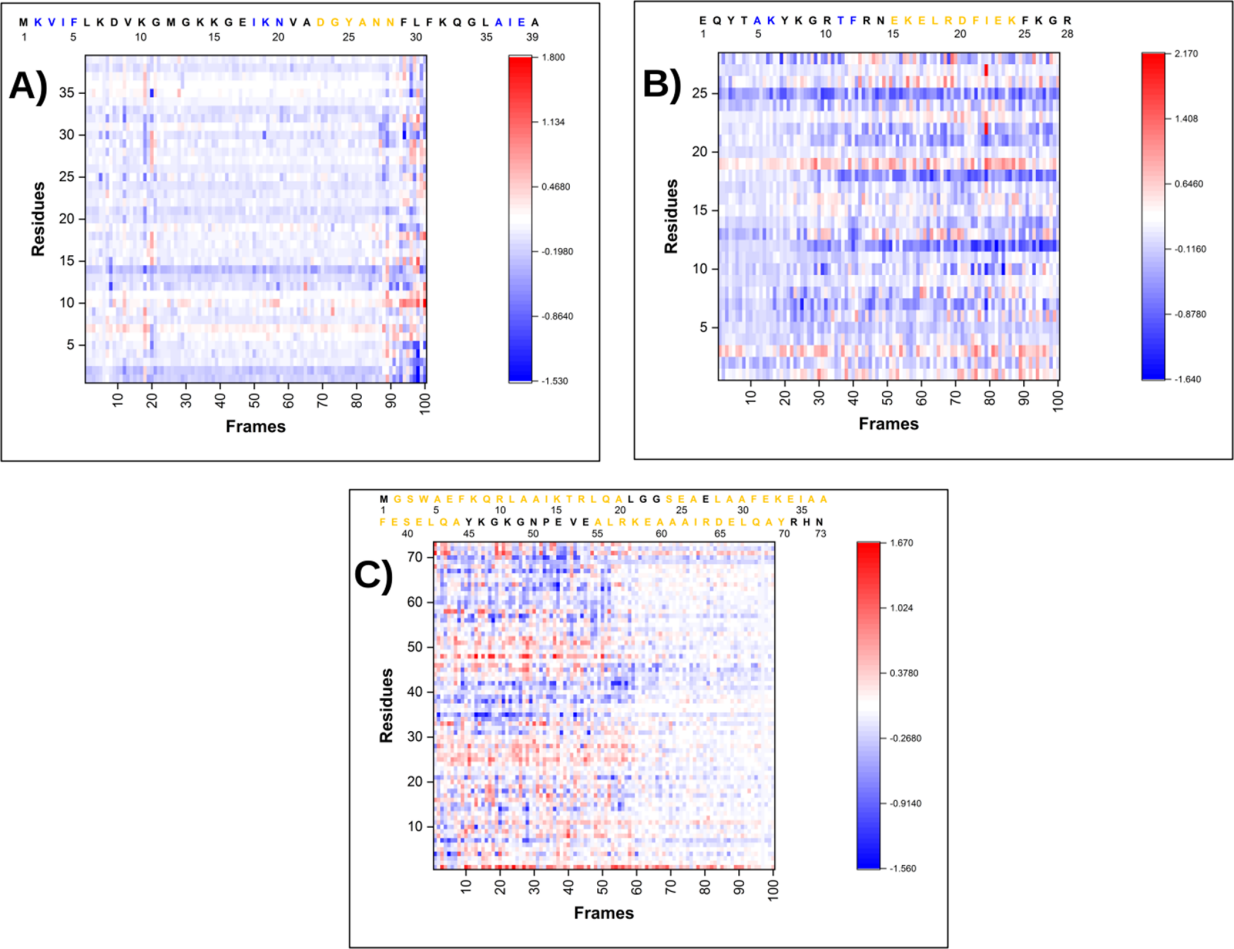
Heatmap of Δ­(η) for (A) NTL9, (B) BBA, and (C) α3D proteins.

When analyzing the data in Figure 6A, we observe that the heatmap of Δη is very similar to the heatmaps generated for the residue-centered RMSD and RMSF (Figures S13a and S14a, respectively), further corroborating that this descriptor follows the protein folding process. In addition, in the region of non-native conformations (frames: 100-91) for the NTL9 protein, some residues are harder than their expected hardness (shown in red) or softer than their expected local hardness (shown in blue), while Δη assumes constant values (close to zero) in the region of native-like structures (frames: 90-1).

This shows an intriguing trend where the local hardness per residue did not increase or decrease consistently. Soft residues do not become softer as the trajectory progresses until they plateau at their expected softness, and neither do hard residues become harder. Rather, this is a more subtle process by which local hardness fluctuates both above and below the expected values of native-like for each residue. These data justify why Faver and Merz^61^ failed to discriminate the native structure from a set of decoy structures when they used reactivity descriptors. The point is not that the better-folded structures have more favorable hard or soft interactions between the residues, but that the local hardness of the residues becomes more stable as the conformation approaches the folded state.

Having more and more stable local hardness, in the context of the working equation that was successful (eq 12), is expected as the atoms in the system are more surrounded by electron density as their distances are shortened. This intensifies the electrostatic and other long-range weak and dispersive forces interactions. The electron density repels itself but also minimizes the repulsion of the positively charged nuclei, leading us to interpret this local descriptor as appropriate to capture all noncovalent/net charge transfer phenomena in these systems.

Figure 6B,C show that Δη_*j*_ behaves similarly for the BBA and α3D proteins as it does for NTL9, although the three proteins have free energy surfaces of distinct roughness, showing that Δη_*j*_ can lead to the identification of important conformations during folding. The data also suggest that local hardness can be used not only in the folding process but also in other conformational search problems.^115–118^

The data in Figure 6 also reveal that some residues showed slight variations in Δη_*j*_ during the folding path, while other residues showed more pronounced changes. The residues Phe-5, Lys-19, and Leu-30 in the NTL9 protein, Figure 6A, showed a considerable increase in their Δη_*j*_ when they transformed from the non-native region to native structures (frames 100-91). Residues Phe-5 and Lys-19 are present in the first two β-sheets, respectively, while residue Leu-30 is located immediately after the α-helix. This suggests that these residues may play important roles in maintaining the stability of these secondary structures.

In BBA, Figure 6B, Δη_*j*_ for residues Phe-12, Leu-18, and Phe-21 showed a sharp increase when the protein reached lower values of RMSD regarding the folding structure (frames 25-1). The residue Phe-12 contributes to the formation of the second β-sheet, while residues Leu-18 and Phe-21 are present in the α-helix.

Finally, in the α3D protein, Figure 6C, residues Phe-7, Phe-31, Ile-35, Phe-38, and Gln-67 showed an increase in Δη_*j*_ when they transitioned from the non-native region to native-like structures (frames 59–100). We observed that residue Phe-7 is present in the first α-helix, residues Phe-31, Phe-35, and Phe-38 are present in the second α-helix, and residue Gln-67 belongs to the third α-helix. As these residues became harder, they played an important role in the formation and maintenance of secondary structures.

#### 3.3.1 Analysis of the Average Local Hardness by Residue Group Types

To verify whether there are local hardness patterns that are intrinsic to each residue, we generated a graph of the local hardness variation by type of residue, considering the three proteins. These results are shown in Figure 7.

**Figure 7:**
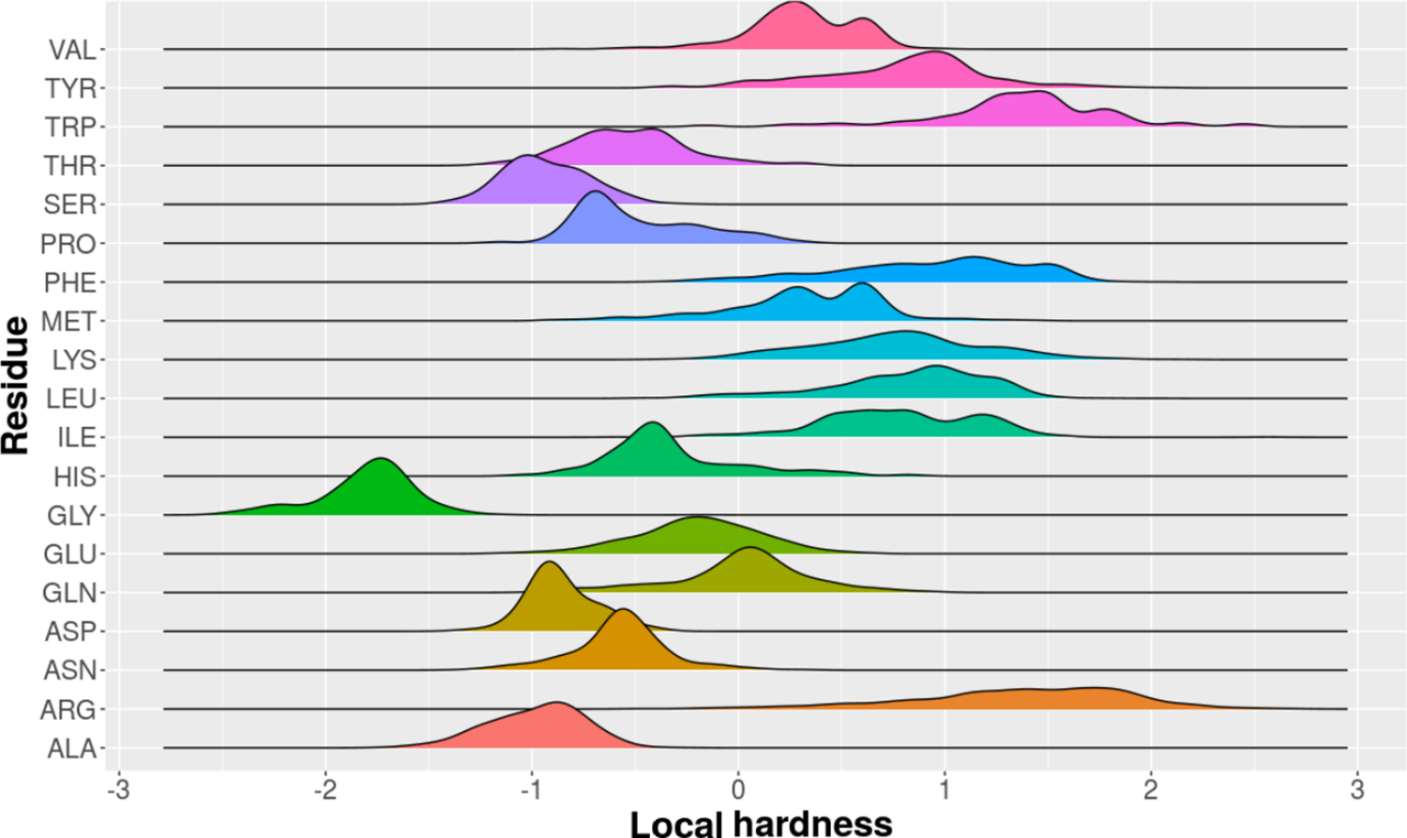
Variations in local hardness by type of residue for proteins NTL9, BBA and α3D. The local hardness of the 19 residues present in the three proteins together was considered.

The local hardness of the residues fluctuates similarly across all three proteins under study. Therefore, we infer that the residues possess intrinsic hardness patterns. The residues of Ala, Asn, Asp, Gly, and Ser are softer, whereas the residues of Arg, Ile, Leu, Lys, Phe, Trp, and Tyr are harder. The residues of Gln, Glu, His, Met, Pro, Thr, and Val have an intermediate local hardness.

Although some residues showed more pronounced changes in the local hardness, such a variation was significant along the entire folding pathway. However, we emphasize that conformations assume constant values when they move to the region of native-like structures, that is, residues assume states of local chemical hardness according to trends presented in the results of Figure 7.

For a more detailed discussion of contributions by residue, we have classified the amino acids into five groups according to their side chain properties: (1) nonpolar aliphatic (Ala, Gly, Ile, Leu, Met, Pro, and Val), (2) aromatic (Phe, Trp, and Tyr), (3) uncharged polar (Asn, Gln, Ser, and Thr), (4) positively charged polar (Arg, His, and Lys), and (5) negatively charged polar (Asp and Glu). In the three proteins studied, we calculated the average values for each group of amino acids in the native-like and non-native regions. The aim was to assess whether there are patterns common to the three proteins according to the group to which the amino acids belong, and whether the position of the amino acid in the primary sequence affects the local hardness variation.

When analyzing Group 1 (Figure S20), we observed some residues that play important roles in protein stability. The residues Met-1, Val-3, and Leu-30 (NTL9); Leu-18 and Ile-22 (BBA); and Ile-14, Ile-35, Leu-42, Leu-63, and Leu-67 (α3D) have high average local hardness and low variability in native-like regions. The other residues did not show significant variations between the two evaluated regions.

In Group 2 (Figure S21), we find that phenylalanine residues have a general propensity to become harder when they move from the non-native region to the native-like region, whereas tyrosines exhibit the opposite behavior. For the NTL9 protein, it is worth noting the large increase in local hardness (from 1.00 to 1.52) for Phe-5 when the protein reaches folded states. This situation does not occur with Phe-29 and Phe-31 residues, which show slight reductions and increases in local hardness, respectively. The reason for this may be that Phe-5 is in the region of formation of the first β-sheet, whereas residues Phe-29 and Phe-30 are in the loop region, as shown in Figure 3B.

This same behavior is observed in the BBA protein, where residues Phe-12 (which belongs to the second β-sheet) and Phe-21 (belonging to the α-helix) presented a much more pronounced increase in the average local hardness within the native-like part of the trajectory compared to Phe-25 (loop region), as shown in Figure 4B.

For the α3D protein, residues Phe-7, Phe-31, and Phe-38 showed an increase in average local hardness when moving from the non-native region to the native-like region, since all of these residues are in α-helices. However, the Phe-38 residue showed a greater increase in the average local hardness. Therefore, it is an important residue for the protein to reach its native state. These data suggest that some secondary structure residues assume greater local hardness when the protein reaches its native state.

In Figure 8, we present in (A) two conformations for the BBA protein, with one structure referring to frame 26 (last frame of the non-native region) and the second representing frame 13 (native state). In (B), we present a bar graph with the average local hardness for the residues of Group 2. Similar graphs for the NTL9 and α3D proteins can be found in Figures S29 and S30.

**Figure 8:**
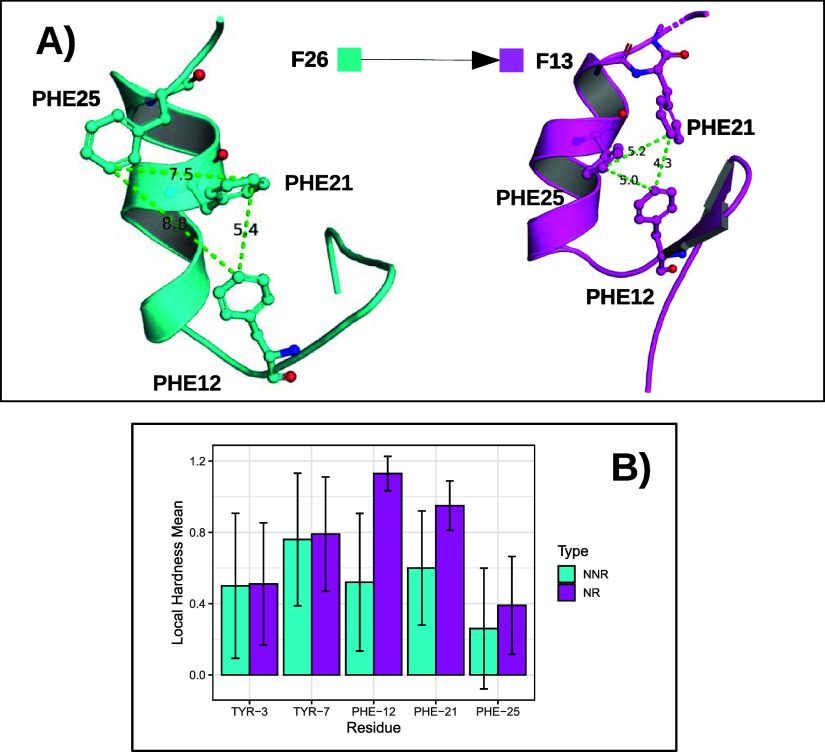
(A) BBA protein conformations for frames 13 and 26 with emphasis on residues Phe-12, Phe-21, and Phe-25. (B) Average local hardness for Group 2 residues of the BBA protein in the non-native region (NNR) and native-like region (NR).

From a structural point of view, residues with greater average local hardness (Phe-12, Phe-21, and Phe-25) have much shorter distances from each other in frame 13 than in frame 26. The same behavior is observed for the residues Phe-5 and Phe-31 of the NTL9 protein and for the residues Phe-7, Phe-31, and Phe-38 of the α3D protein, as shown in Figures S29 and S30, respectively. This suggests that residues that have a higher average local hardness in the native-like region control the protein folding process.

In addition, the vertical lines displayed around the average local hardness values in Figure 8B represent the intrinsic local hardness variability along the RNS/LNS trajectory of each residue, reflecting the QCMD fluctuations inherent to each conformational state, showing that the values in the native-like structure are higher and more stable for residues Phe-12, Phe-21, and Phe-25. This variability represents a fundamental feature of the protein’s electronic structure. In the native state, we observed a significant reduction in local hardness fluctuations, showing greater stability of the electronic structure at the residue level. In contrast, in the non-native state, the dispersion of values is more pronounced, reflecting the reduced structural organization of the electronic framework compared to the functional conformation of the native state.

When analyzing Group 3 (Figure S22), we found that the residues (Asn, Gln, Thr, and Ser) tended to have very low average local hardness values (most with negative values) and that in the non-native region, they had average values of local hardness slightly higher than those in the native region. For the NTL9 protein, we verified that residues Asn-27 and Asn-28 (which belong to the α-helix) showed a greater decrease in the average local hardness when they moved to the native region. The same behavior is observed for the BBA protein with the Asn-14 residue (which belongs to the α-helix). Thus, we suggest that these residues play an important role in the formation of the α-helix when the NTL9 and BBA proteins enter the native state. For protein α3D, we observed that residues Asn-50, Asn-73 (loops), Thr-4, Ser-24, and Ser-40 (α-helices) had lower average local hardness values in the native-like region.

For Group 4 (Figure S23), we see that, in general, for all proteins studied, neither lysines nor arginines show significant variations in the average local hardness when moving from the non-native region to the native-like region. Arginines have an average local hardness that far exceeds that of lysines, except for Arg-28. This is because Arg-28 is the last residue in the sequence (loop segment) and is therefore more flexible.

Interestingly, in the NTL9 protein, we verified sudden variations in the average local hardness of residues Lys-10 and Lys-19; however, they occurred in opposite directions. Lys-19 becomes harder when it reaches the folded state, while Lys-10 becomes softer. We initially verified from the primary sequence (Figure 3B) that Lys-2 (in the first β-sheet) and Lys-7 (residue close to the first β-sheet) have a higher average local hardness in the native region. We also noticed a sharp reduction in the average local hardness of Lys-10 (loop away from the β-sheets). The average local hardness increases again in the Lys-14 and Lys-15 residues (residues close to the second β-sheet), with the maximum local hardness value observed for the Lys-19 residue (residue in the second β-sheet). Thus, for the NTL9 protein, we suggest that the average local hardness tends to be higher in the β-sheets because of an inductive hardness effect on the residues close to these secondary structures, since these residues are positively charged.

For the α3D protein, we observed that the Lys-46 and Lys-48 residues (loop segment) showed a greater reduction in average local hardness when moved to the native-like region. These data suggest that the loop segment plays an important role in the α3D protein folding process.

In Figure 9, we observed that the residues with higher values of average local hardness in the native-like region (Lys-2 and Lys-19) showed greater structural variations when passing from the unfolded state to the native state (Figure 9 A). The Lys-10 and Lys-32 residues, which had low average local hardnesses in the native-like region, did not present significant structural changes. These results corroborate those observed in the analysis of residues in Group 2, in which residues with a higher average local hardness are the most important in the protein folding process.

**Figure 9:**
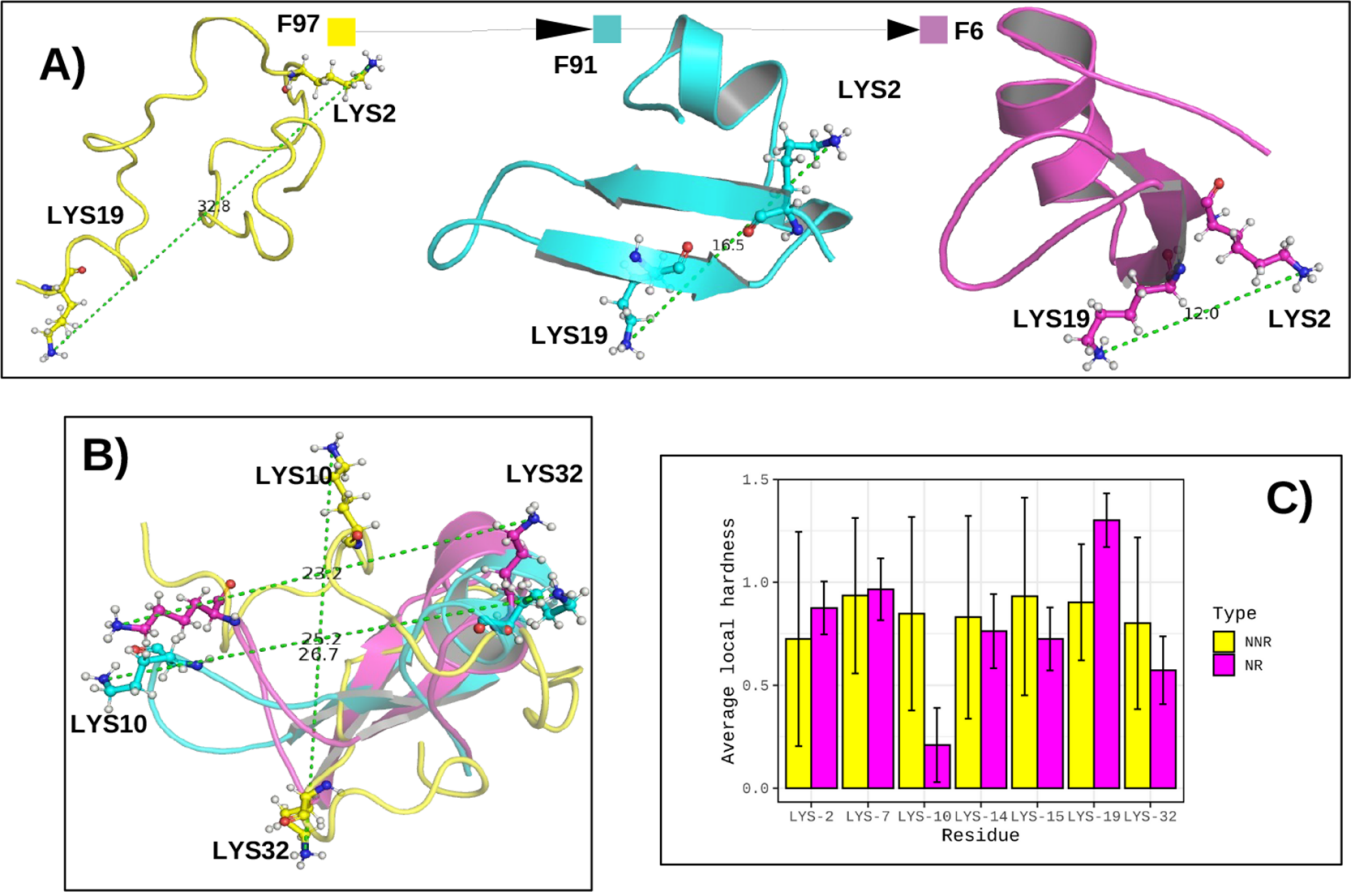
In (A) NTL9 protein conformations for frames 97, 91, and 6 with an emphasis on residues Lys-2 and Lys-19. In (B) aligned structures of frames 97, 91, and 6 with an emphasis on residues Lys-10 and Lys-32. In (C) average local hardness for group-4 residues in the nonnative region (NNR) and native-like region (NR).

When analyzing Group 5 (Figure S24), we found that for the proteins NTL9 and BBA, glutamate and aspartate residues showed negative average local hardnesses, showing similar values in non-native and native-like regions. It should be noted that aspartate residues had average local hardness values lower than those of glutamate residues in the three proteins studied. For the α3D protein, we found that the Glu-25 and Glu-27 residues have much lower average local hardness in the native-like region, whereas the Glu-34 and Glu-39 residues have much higher average local hardness in that same region. This suggests that these residues are important in the α3D protein folding process.

## 4 Conclusions

In this study, we evaluated aspects of the electronic structure of three fast-folding proteins (NTL9, BBA, and α3D) through QCMDs obtained by DFT-D3 and PM7 methods during their RNS/LNS pathways. The analyses of local QCMDs revealed important aspects of the role of the electronic structure throughout protein folding. We observed that the local hardness and electron density become constant as the protein approaches the native state, which correlates very well with RMSF and RMSD per residue. In this way, it is possible to understand aspects of the free energy surface and to predict whether the conformations are moving toward the native state or not by evaluating the local hardness. Although there are several techniques capable of distinguishing native-like states from non-native states, the analysis of local hardness differs because it does not depend on a previously known structure. The Δη_*j*_ (Figure 6) produced a heatmap very similar to those obtained with RMSF and RMSD data per residue, further demonstrating the correlation between local hardness and structural properties. In addition, the results also correlate with the formation of secondary structures, as seen in the DSSP analyses (Figures S15–S17).

We suggest that residues with a higher average local hardness during the folding pathway are those that control the process and, therefore, should have their properties individually monitored throughout protein folding. We validated all results through DFT-D3, but we emphasize that the calculations using the semiempirical PM7 method showed the same patterns; therefore, it is possible to evaluate all descriptors with PM7 with similar quality and with lower computational cost.

Finally, the usage of quantum chemical molecular descriptors, especially reactivity descriptors, calculated through the semiempirical PM7 method, can unlock the boundaries of the study of macromolecular systems, allowing the study of electronic structures of biomolecules using low-cost processors. This is evident based on the knowledge that the PM7/MOZYME/COSMO single-point calculation for the NTL9 protein on a single notebook processor (Intel Core i5-7300HQ 2.50 GHz) was approximately 1700 times faster than the B3LYP-D3 calculation run on an NVIDIA Tesla K40 GPU for the same structure.

## Supplementary Material



## Data Availability

Data used in this study are in the open access online repository Zenodo (https://zenodo.org/record/7386905#.Y4ntpHbMJPY, DOI: 10.5281/zenodo.7386905). All data is zipped into the all_data_v1.0.zip file. This zipped file contains: (i) pdb files for each frame retrieved from RNS/LNS trajectories, (ii) examples of PRIMoRDiA input files for calculating global and local descriptors, (iii) examples of PRIMoRDiA output files with calculated global and local descriptors, (iv) terachem and mopac input files, (v) terachem and mopac output files, and (vi) Excel files containing data for all local descriptors per residue for each frame and all proteins.

## Notes

(i) Global QCMDs obtained by using the PM7 semiempirical method, (ii) global QCMDs obtained by using the B3LYP-D3 method, (iii) structural descriptors, (iv) local QCMDs obtained by using the B3LYP-D3 method, and (v) local QCMDs obtained by using the PM7 semiempirical method (PDF)
