# Supplementary material for: The Role of the Electronic Structure during Protein Folding through Electron Density-Based Quantum Chemical Descriptors
Source: ACS Omega. 2026 Jan 10;11(3):3796–811. doi: 10.1021/acsomega.5c05968 (PMC12854502; doi:10.1021/acsomega.5c05968)
Supplement: Supplementary file 1 [file ao5c05968_si_001.pdf]

Supplementary Information: The Role of the Electronic Structure during Protein Folding through Electron Density-Based Quantum Chemical Descriptors

Acassio Rocha-Santos,[†] Igor Barden Grillo,[†] Gabriel Aires Urquiza-Carvalho,[‡] and Gerd Bruno Rocha[*,†]

†*Department of Chemistry, Federal University of Paraíba, Cidade Universitária, João Pessoa - PB, 58051-900, Brazil.*

‡*Department of Chemistry, Federal University of Pernambuco, Cidade Universitária, Recife - PE, 50670-901, Brazil.*

E-mail: gbr@quimica.ufpb.br

Phone: +55-83-3216-7437. Fax: +55-83-3216-7437

1 Global QCMDs obtained via the PM7 Semiempirical Method

Single-point calculations of 100 representative conformations (for each of the proteins studied) of the folding/unfolding pathway were performed, using the PM7 method and the implicit solvent model COSMO with the MOPAC program. Through these data, we obtain the values of the total energy (E_{TOT}) and the heat of formation (ΔH_f). Using the PRIMoRDiA program, we obtained the following global reactivity descriptors: ionization potential (IP), electron affinity (EA), chemical potential (μ), chemical hardness (η), chemical softness (S), electrophilicity (ω) and maximum number of electrons (n_{Max}). In addition, we perform calculations of the structural descriptors RMSD-Cα and fraction of native contacts (Q) to assess whether these present a correlation with the global reactivity descriptors or with values of (E_{TOT}) and (ΔH_f). Figures S1, S2, and S3 below show the correlation data between all descriptors.

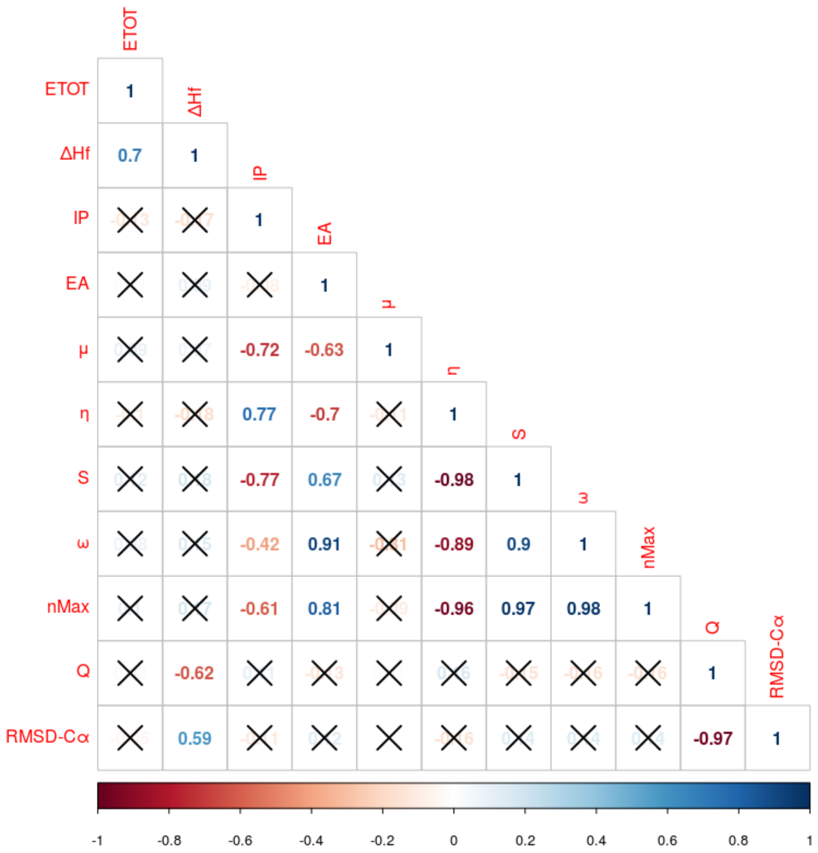

Figure S1: Correlation map between the global reactivity descriptors, E_{TOT} and ΔH_f (obtained via PM7) and the structural descriptors Q and RMSD-Cα for the first tICA coordinate of NTL9.

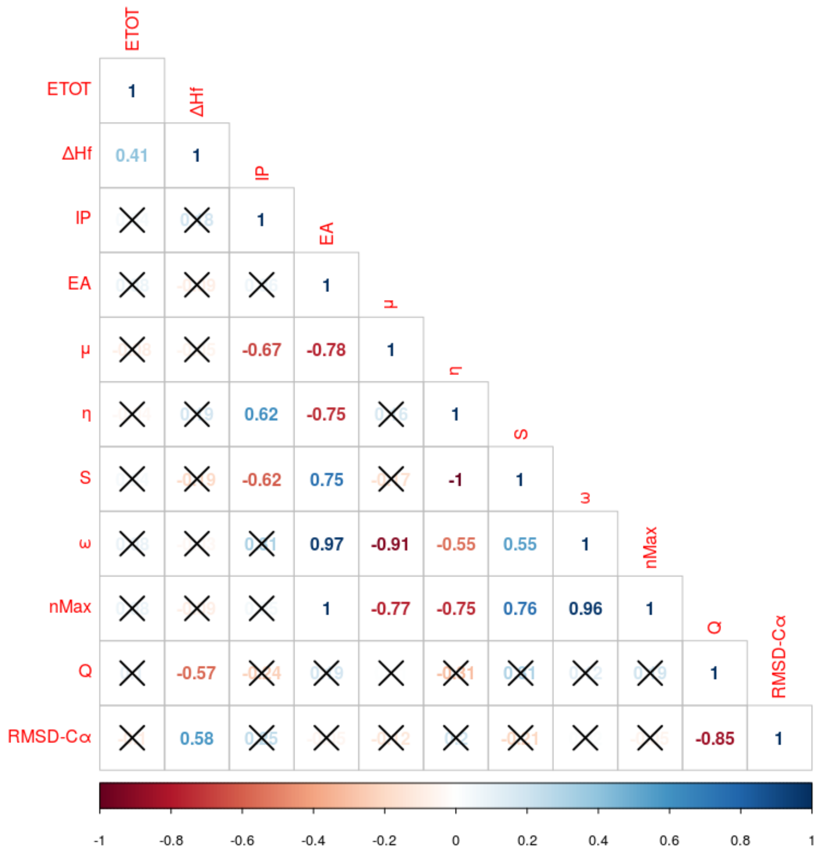

Figure S2: Correlation map between the global reactivity descriptors, E_{TOT} and ΔH_f (obtained via PM7) and the structural descriptors Q and RMSD-Cα for the first tICA coordinate of BBA.

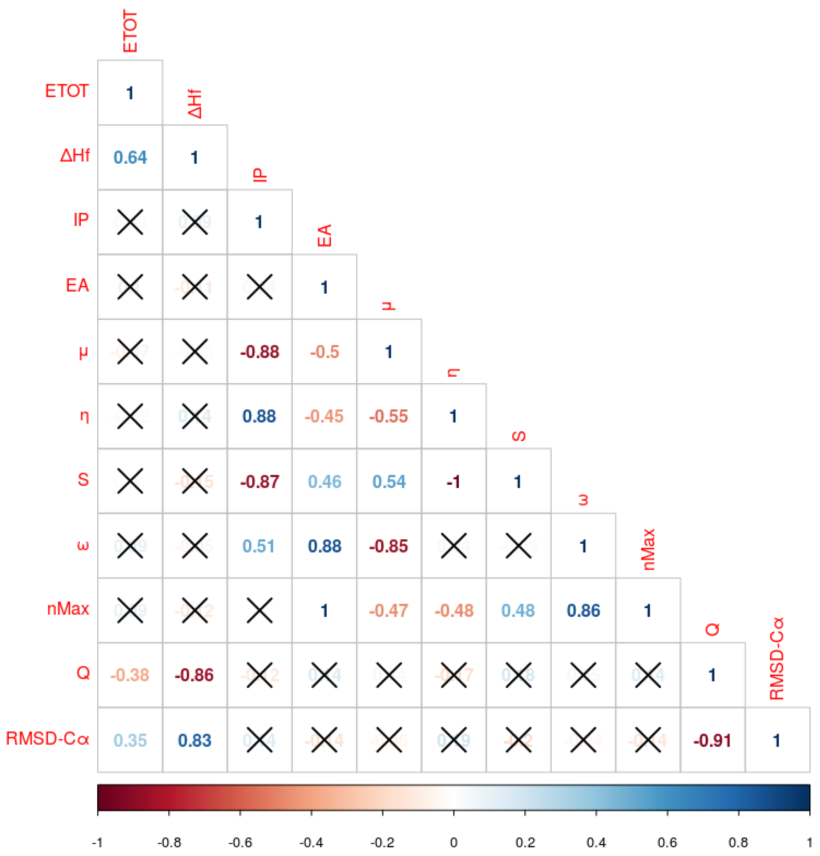

Figure S3: Correlation map between the global reactivity descriptors, E_{TOT} and ΔH_f (obtained via PM7) and the structural descriptors Q and RMSD-Cα for the first tICA coordinate of α3D.

An useful way to expand our analysis is to plot, in addition to the pairwise correlation map, a histogram with the density line and trend line between the variables of our data. In Figures S4, S5, and S6, we present the histogram of all variables in our dataset for proteins NTL9, BBA and α 3D, respectively.

On the main diagonal of this graph, histograms with a density line are presented for each of the variables. In the upper triangle in this graph grid, all comparisons are made between each pair of variables in which the value in bold corresponds to the correlation coefficient R. The asterisks correspond to the p value as follows: 3 asterisks ($p < 0.001$), 2 asterisks ($0.001 < p < 0.01$), 1 asterisk ($0.01 < p < 0.05$), 1 point ($0.05 < p < 0.10$) and case ($p > 0.10$). The correlation coefficient appears without any asterisks or dots. In the lower triangle, there are

plots that consider each pair of variables defined by the intersection of the row and column.

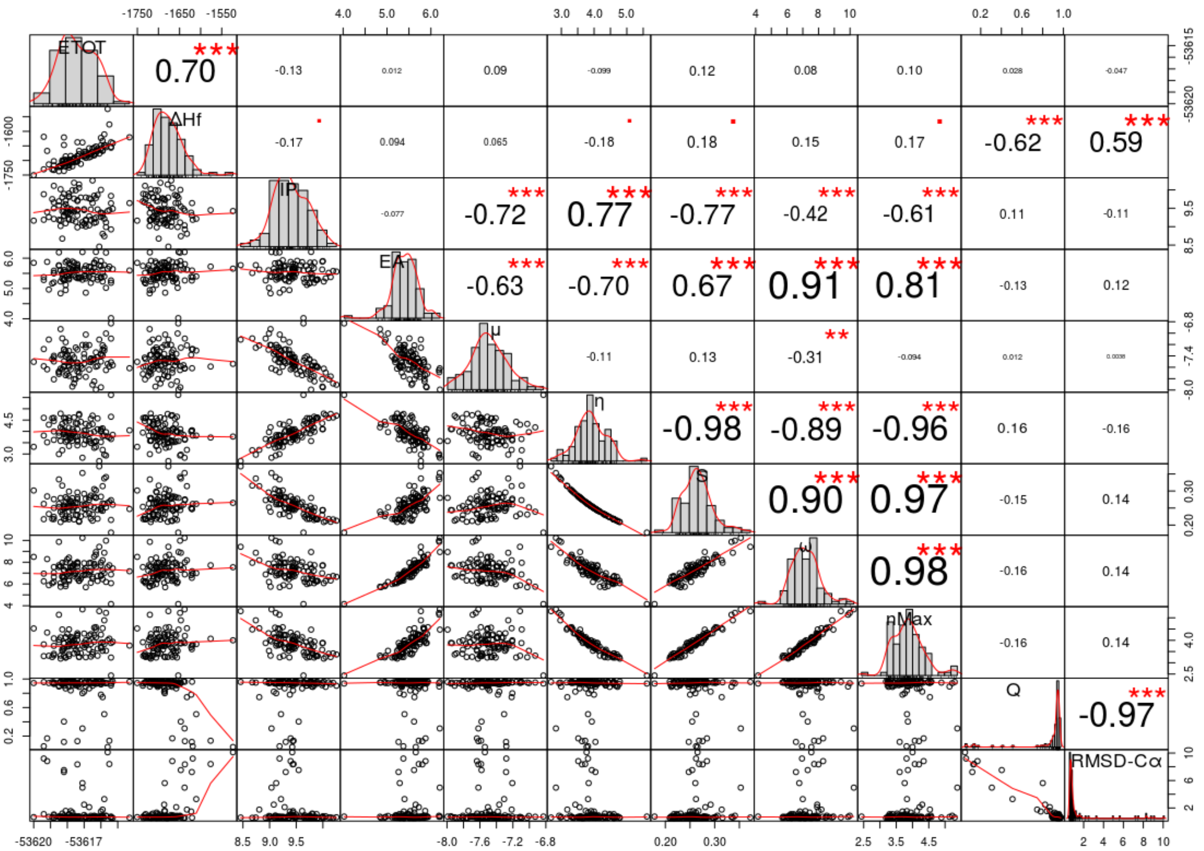

Figure S4: Histogram with correlation, density line, and trend line referring to unfolding coordinate data for the NTL9 protein. Global descriptors were obtained via PM7.

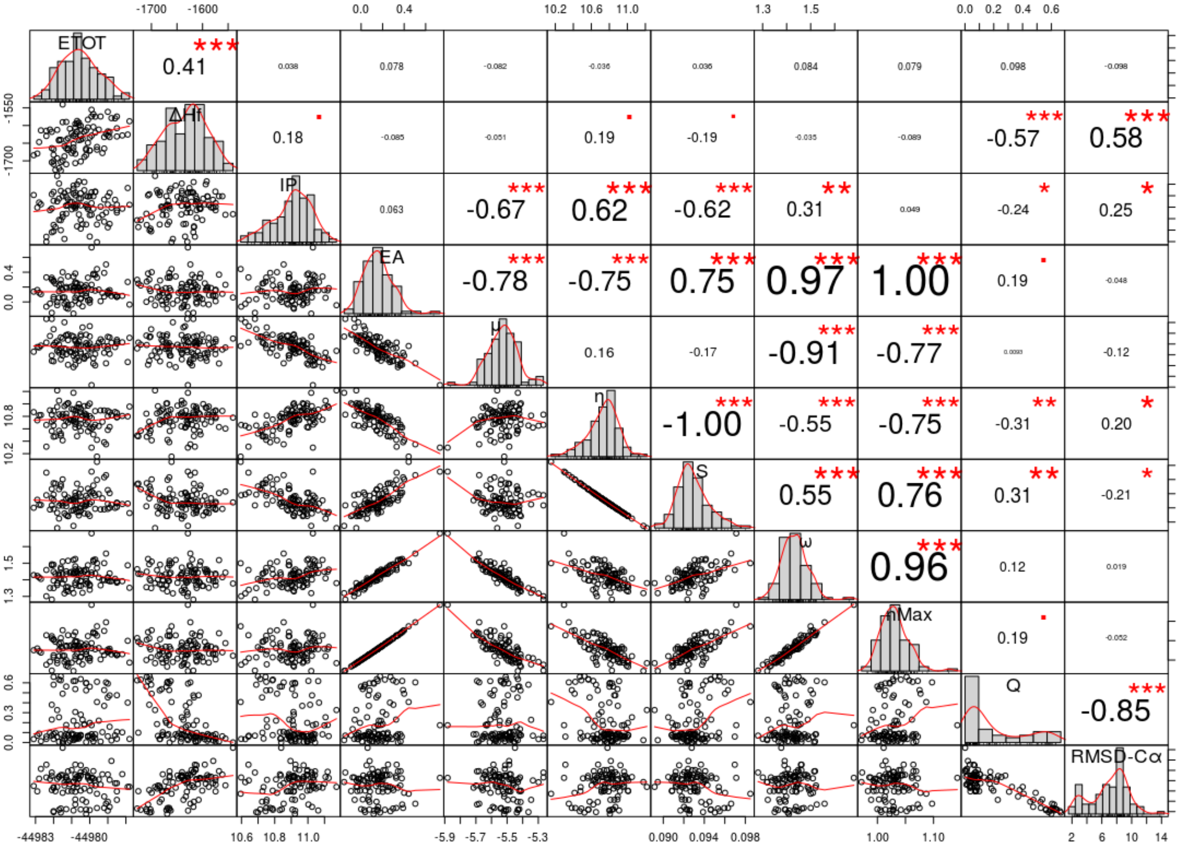

Figure S5: Histogram with correlation, density line, and trend line referring to unfolding coordinate data for the BBA protein. Global descriptors were obtained via PM7.

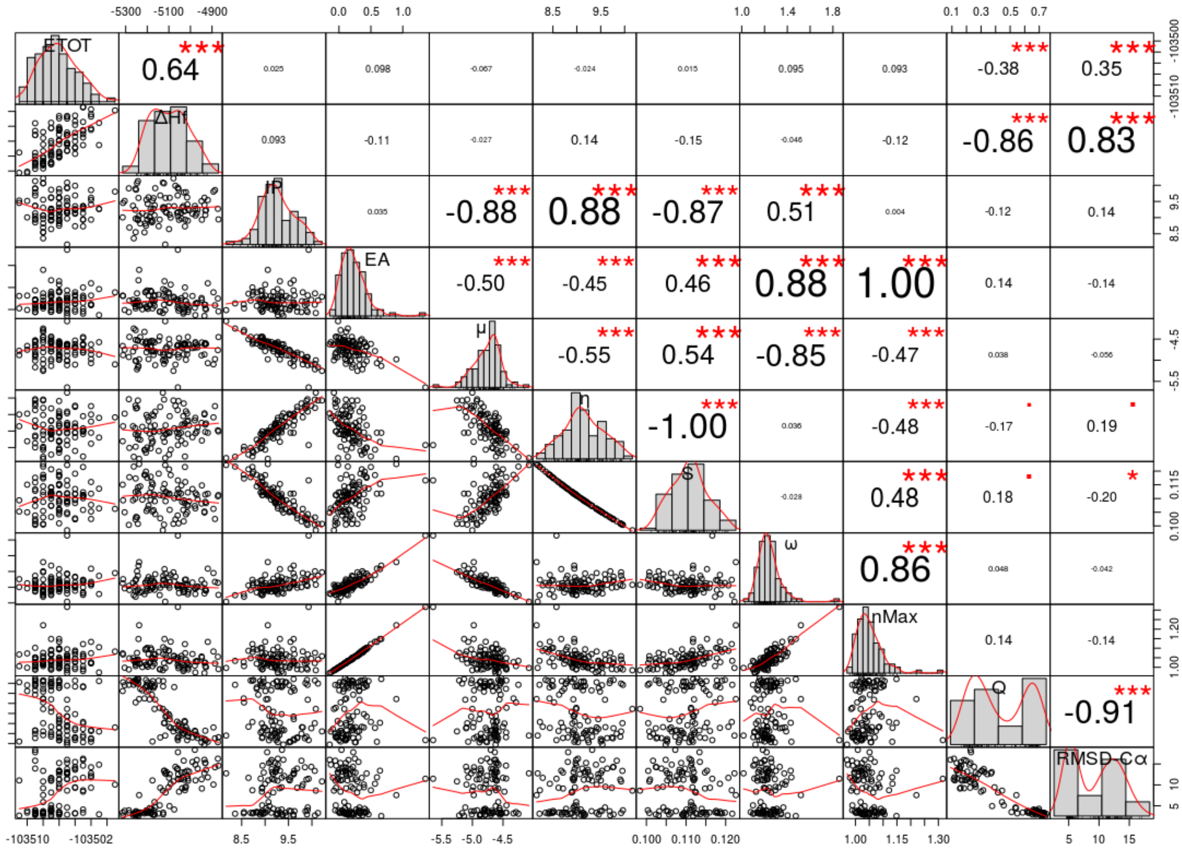

Figure S6: Histogram with correlation, density line, and trend line referring to folding coordinate data for the α3D protein. Global descriptors were obtained via PM7.

2 Global QCMDs obtained via the DFT-D3 Method

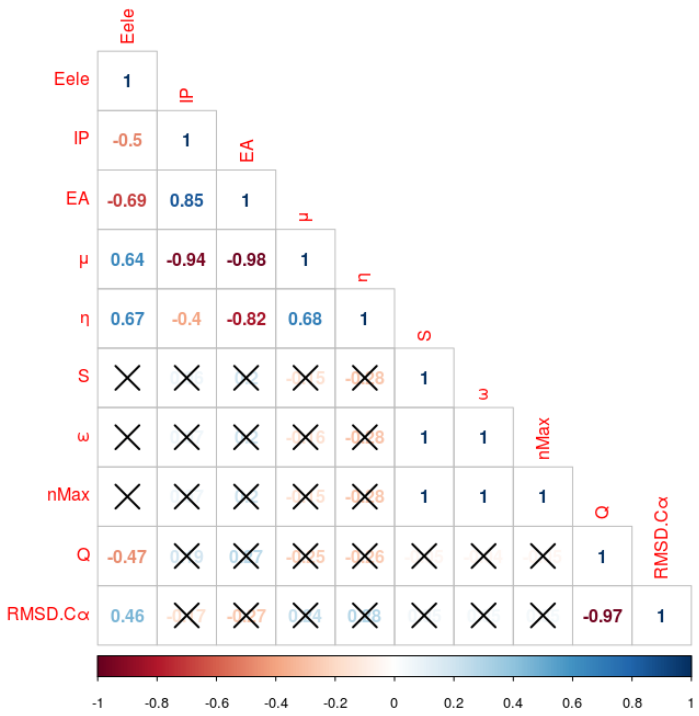

Figure S7: Correlation map between the global reactivity descriptors, E_{ele} (obtained via DFT-D3), and the structural descriptors Q and RMSD-Cα for the first tICA coordinate of NTL9.

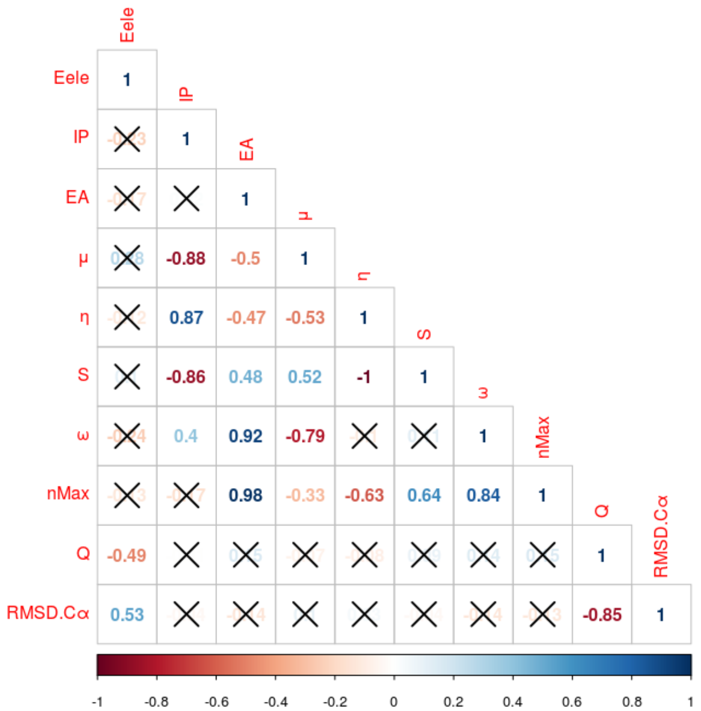

Figure S8: Correlation map between the global reactivity descriptors, E_{ele} (obtained via DFT-D3), and the structural descriptors Q and RMSD-Cα for the first tICA coordinate of BBA.

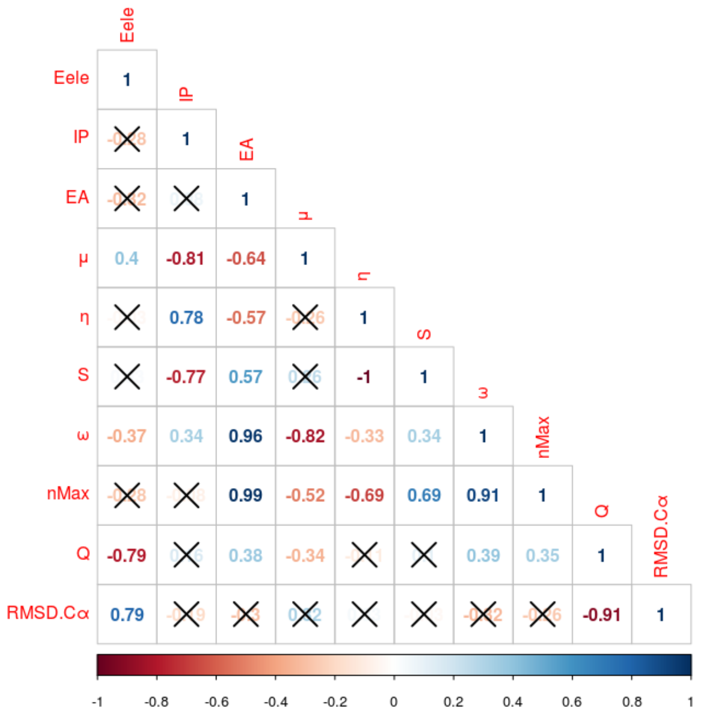

Figure S9: Correlation map between the global reactivity descriptors, E_{ele} (obtained via DFT-D3), and the structural descriptors Q and RMSD-Cα for the first tICA coordinate of α3D.

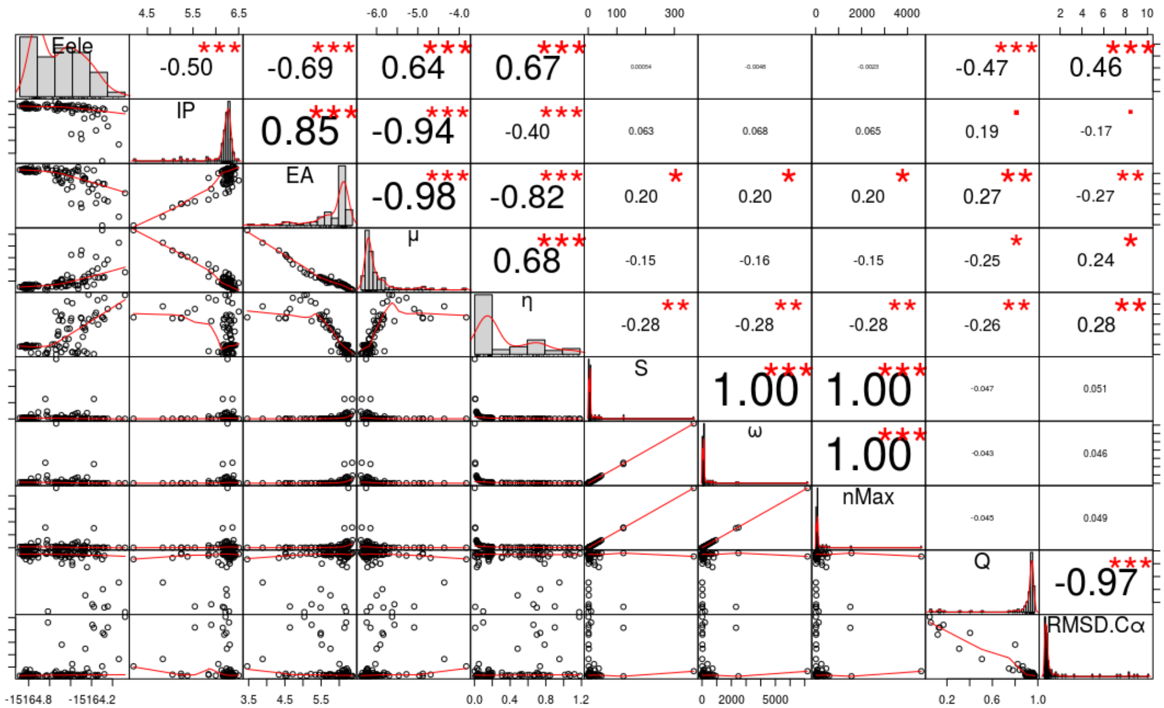

Figure S10: Histogram with correlation, density line and trend line referring to unfolding coordinate data for the NTL9 protein. Global descriptors were obtained via DFT-D3.

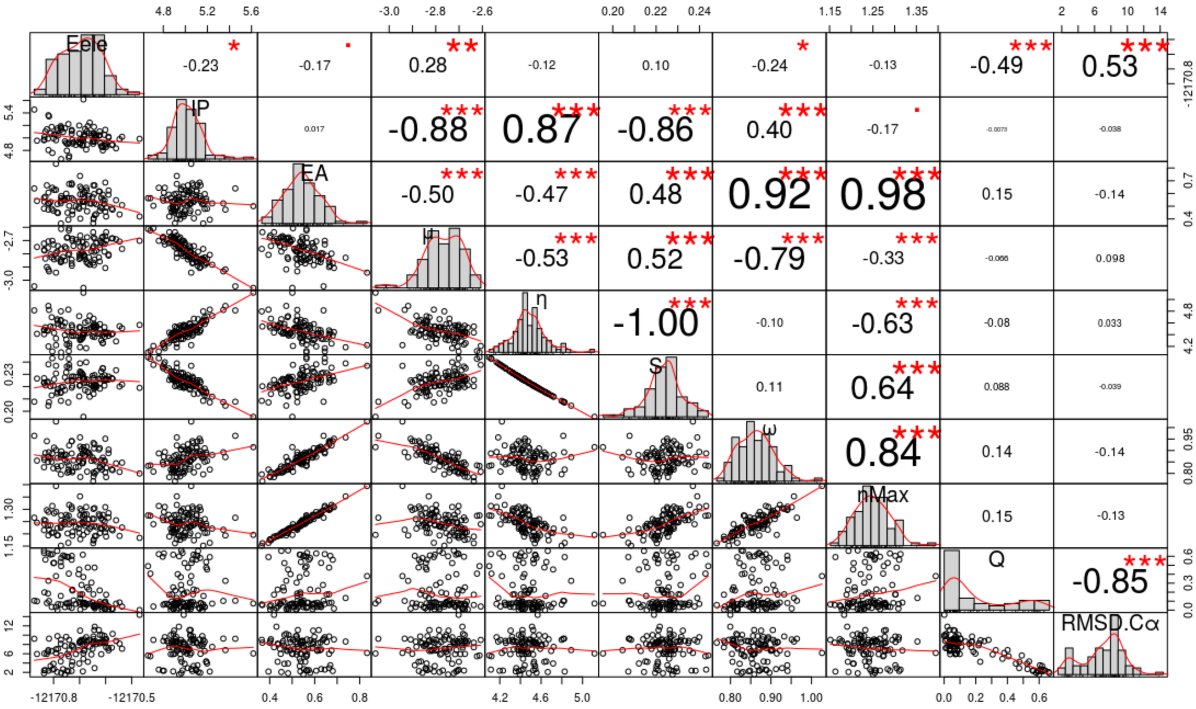

Figure S11: Histogram with correlation, density line and trend line referring to unfolding coordinate data for the BBA protein. Global descriptors were obtained via DFT-D3.

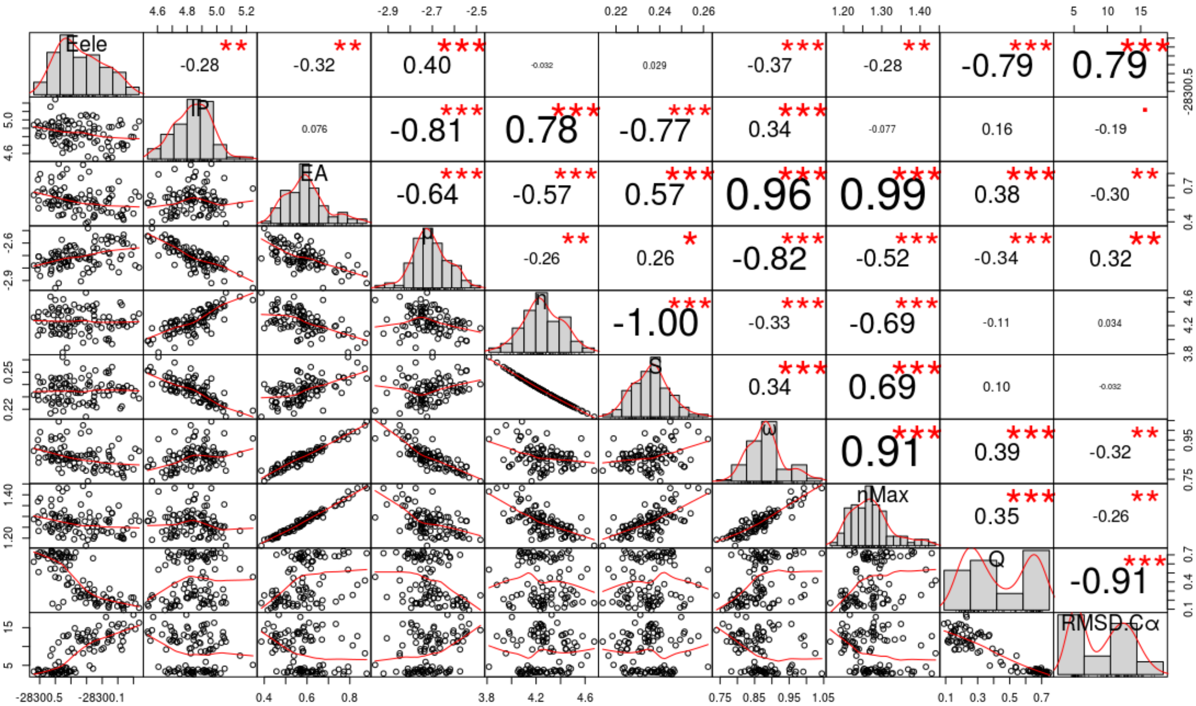

Figure S12: Histogram with correlation, density line and trend line referring to folding coordinate data for the α3D protein. Global descriptors were obtained via DFT-D3.

3 Structural Descriptors

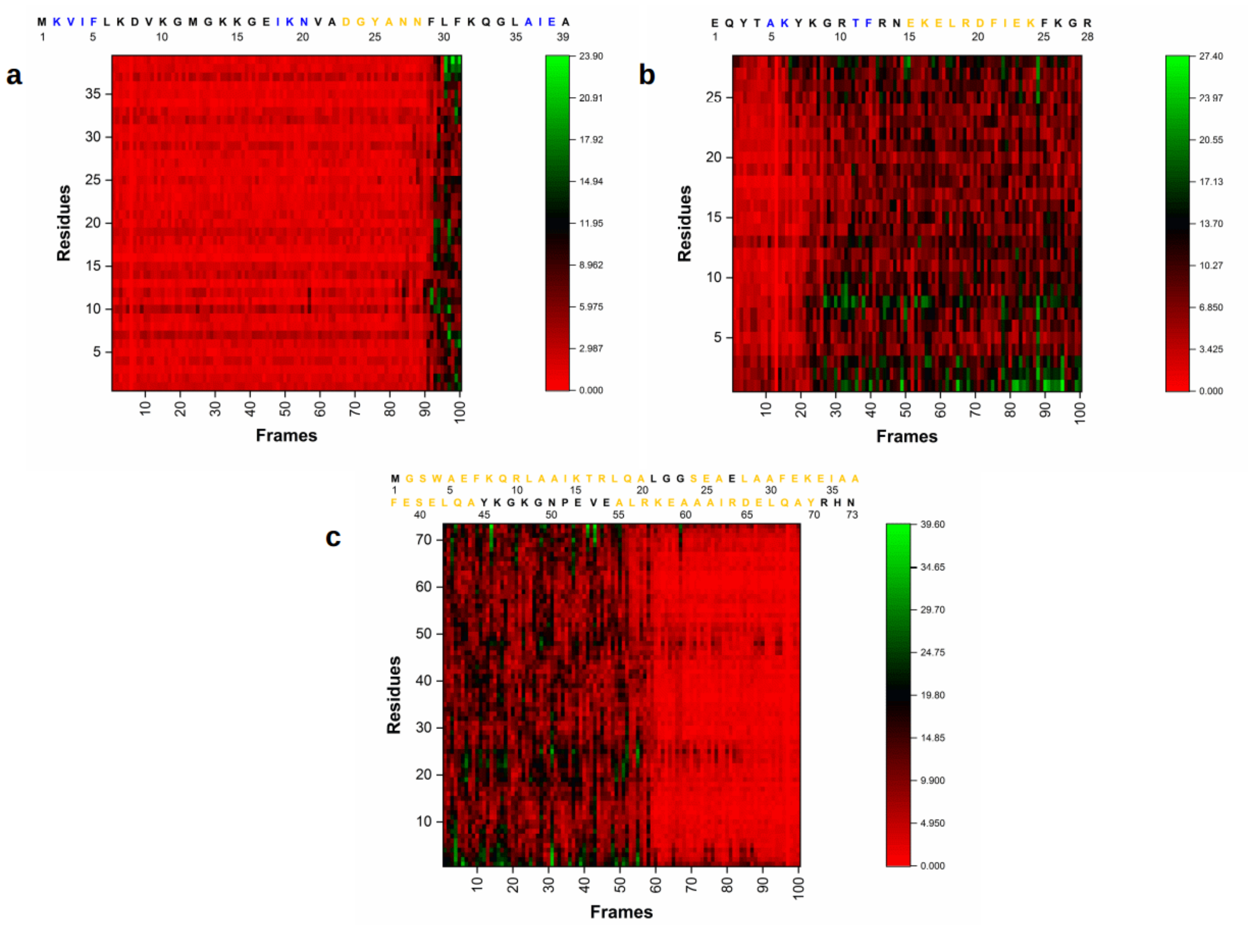

Figure S13: RMSD-Cα per residue for the proteins: (a) NTL9, (b) BBA and (c) α3D.

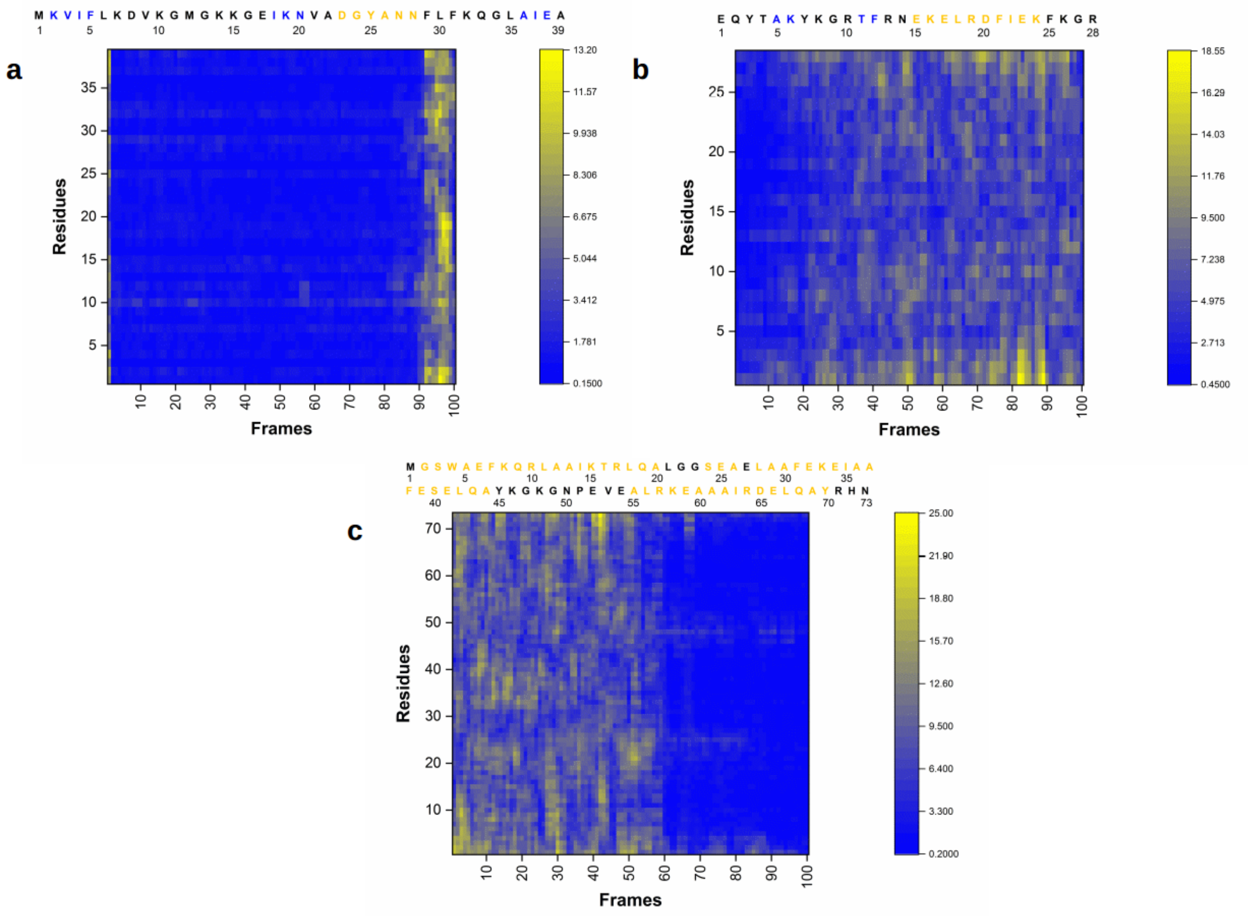

Figure S14: RMSF per residue for the proteins: (a) NTL9, (b) BBA and (c) α3D.

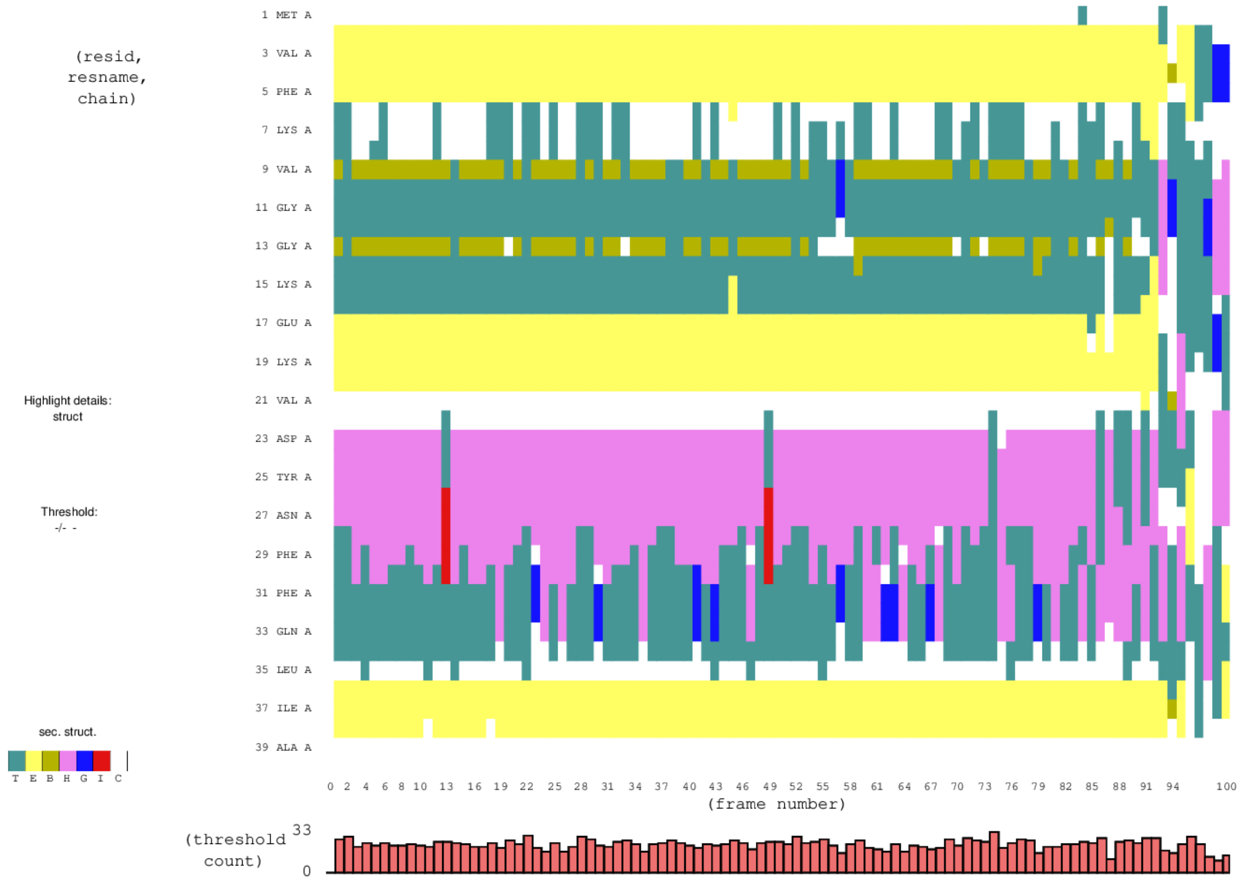

Figure S15: NTL9 protein secondary structure graph.

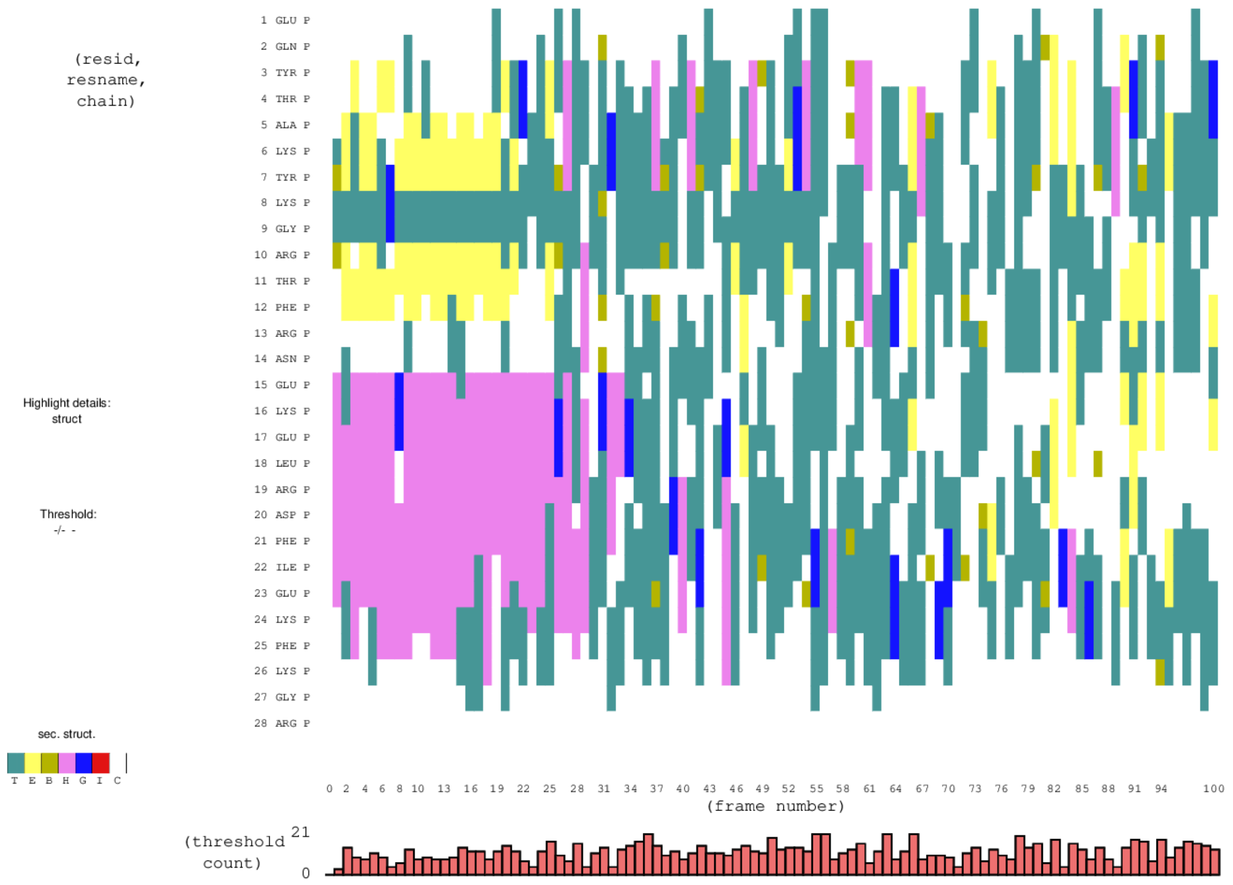

Figure S16: BBA protein secondary structure graph.

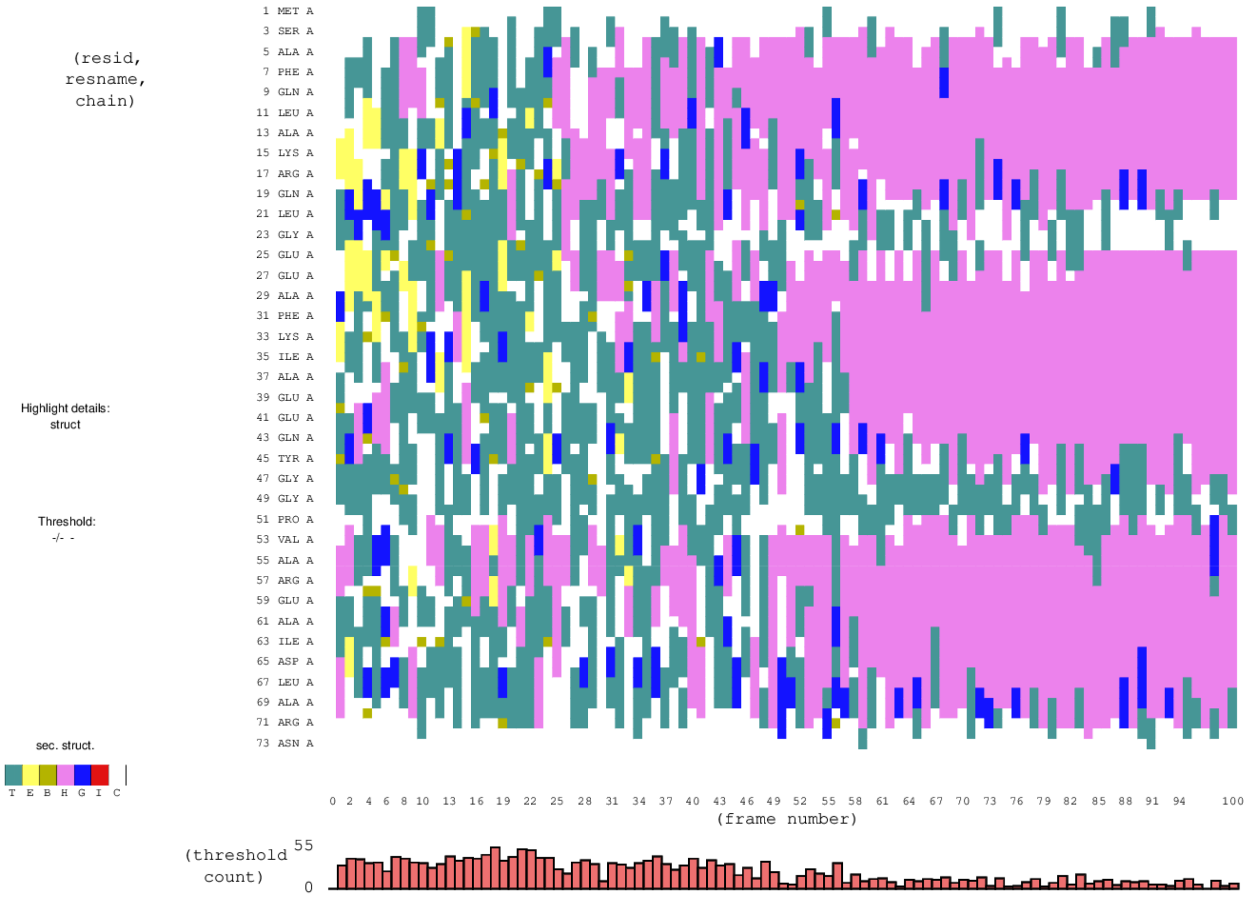

Figure S17: α3D protein secondary structure graph.

4 Local QCMDs obtained via DFT-D3 Method

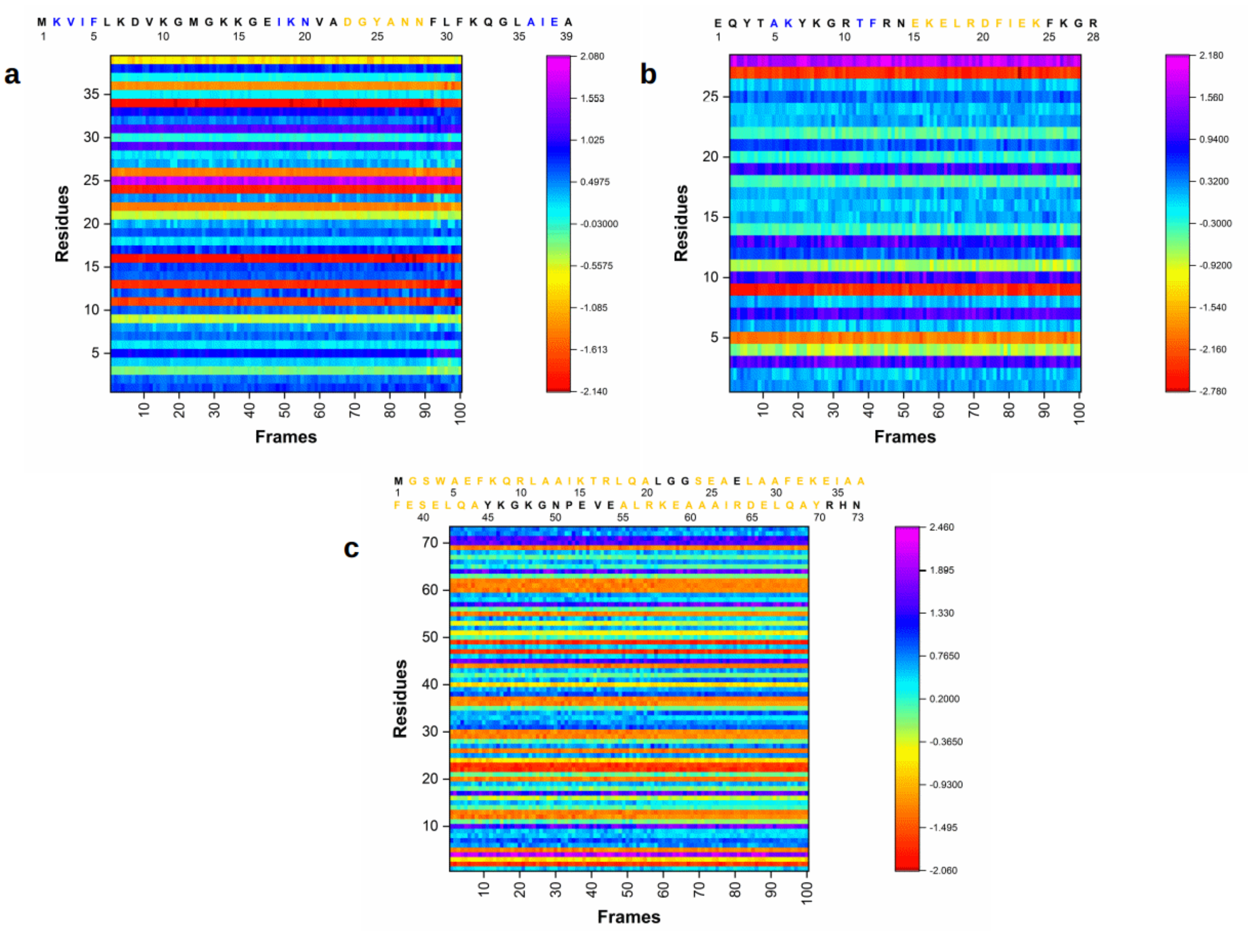

Figure S18: Local electron density heatmap for the proteins: (a) NTL9, (b) BBA, and (c) α3D obtained via the DFT-D3 method.

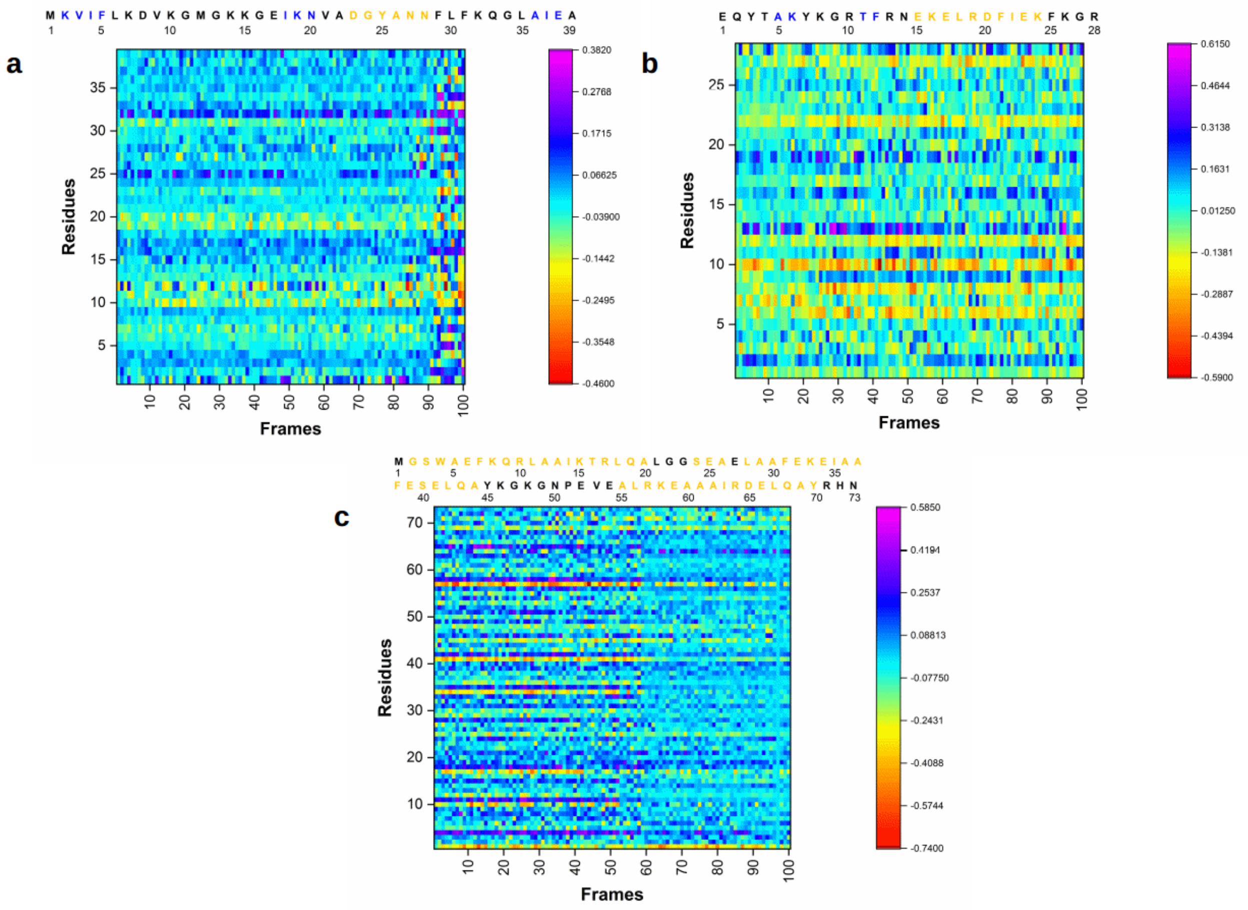

Figure S19: Variation in local electron density ($\Delta\rho$) heatmap for the proteins: (a) NTL9, (b) BBA, and (c) α3D obtained via the DFT-D3 method.

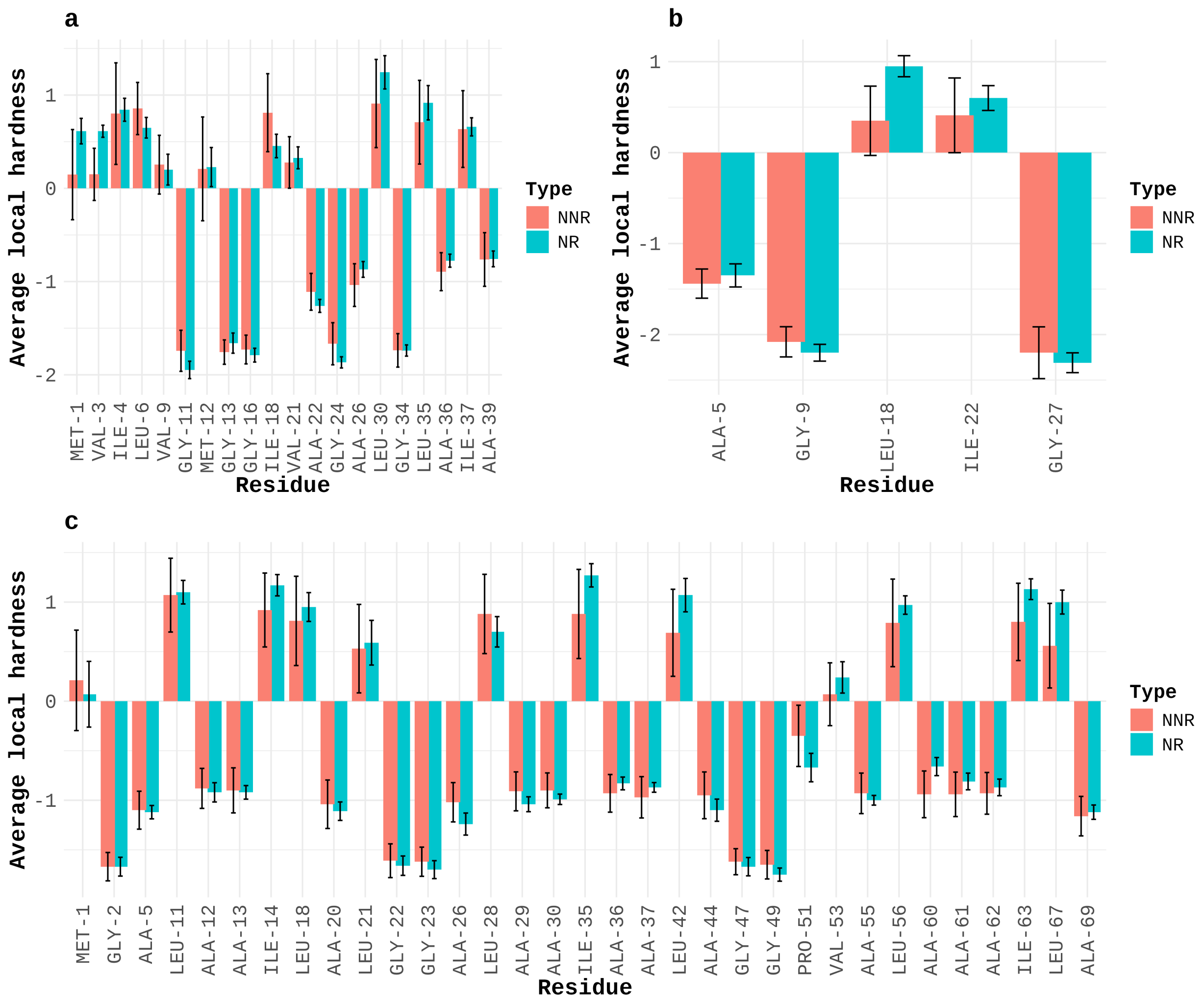

Figure S20: Average local hardness of nonpolar aliphatic residues (Group 1) for proteins: (a) NTL9, (b) BBA, and (c) α3D obtained via the DFT-D3 method.

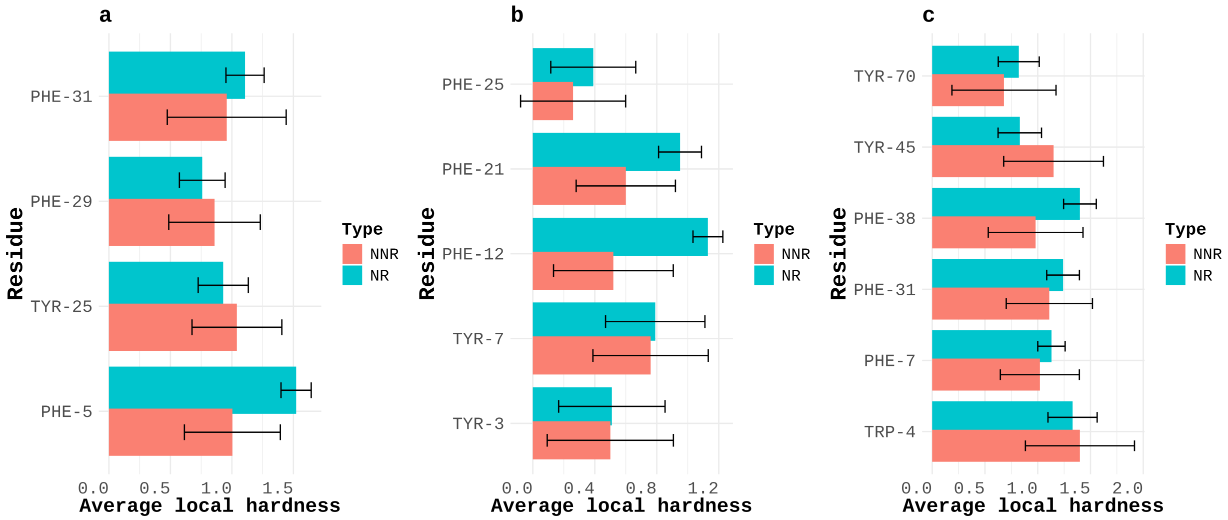

Figure S21: Average local hardness of aromatic residues (Group 2) for proteins: (a) NTL9, (b) BBA, and (c) α3D obtained via the DFT-D3 method.

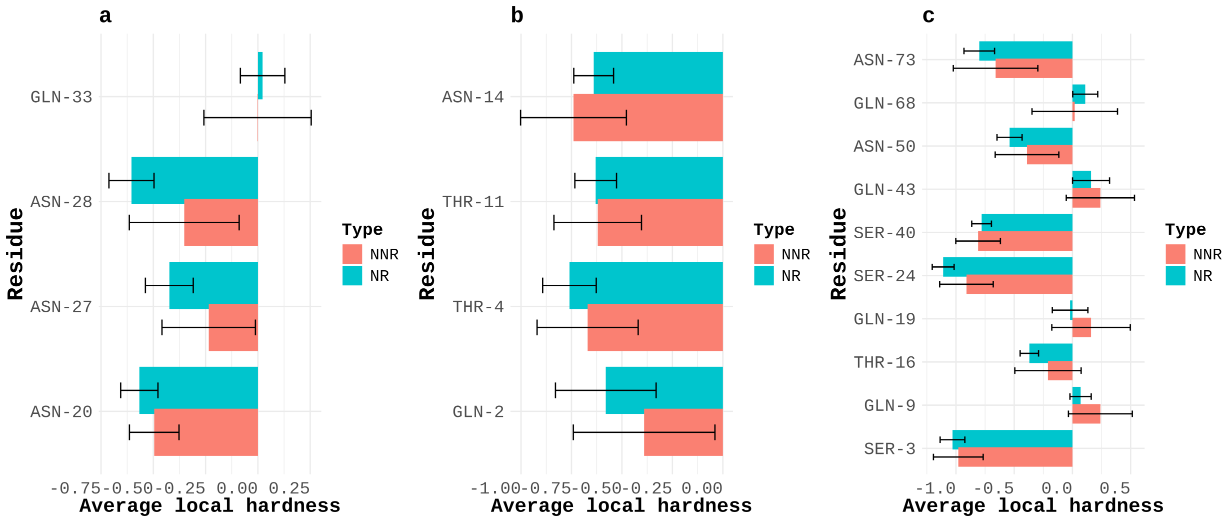

Figure S22: Average local hardness of uncharged polar residues (Group 3) for proteins: (a) NTL9, (b) BBA, and (c) α3D obtained via the DFT-D3 method.

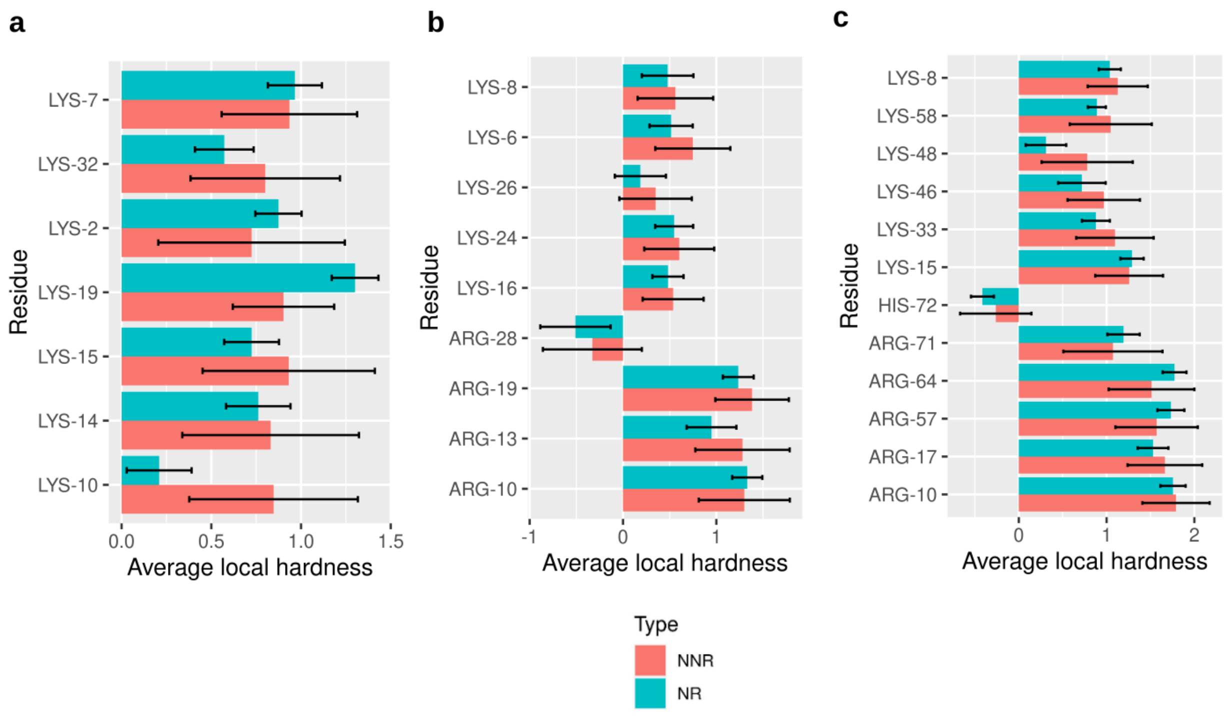

Figure S23: Average local hardness of positively charged polar residues (Group 4) for proteins: (a) NTL9, (b) BBA, and (c) α3D obtained via the DFT-D3 method.

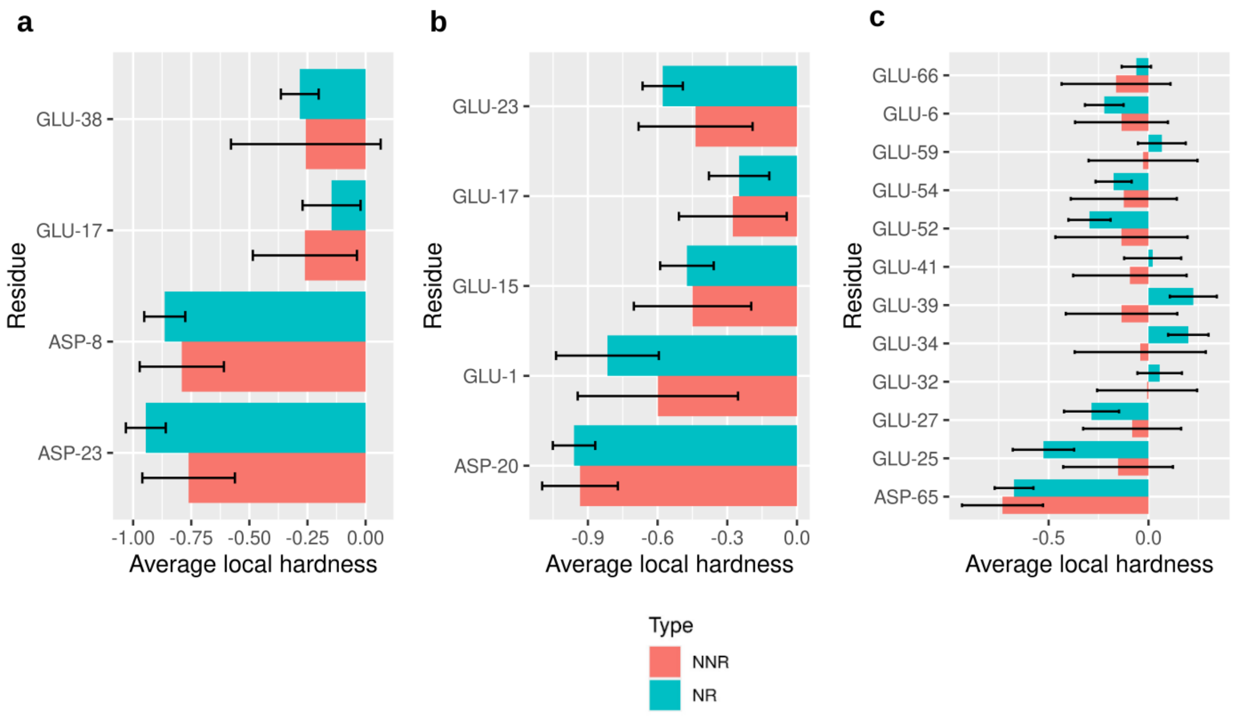

Figure S24: Average local hardness of negatively charged polar residues (Group 5) for proteins: (a) NTL9, (b) BBA, and (c) α3D obtained via the DFT-D3 method.

5 Local QCMDs obtained via the PM7 Semiempirical Method

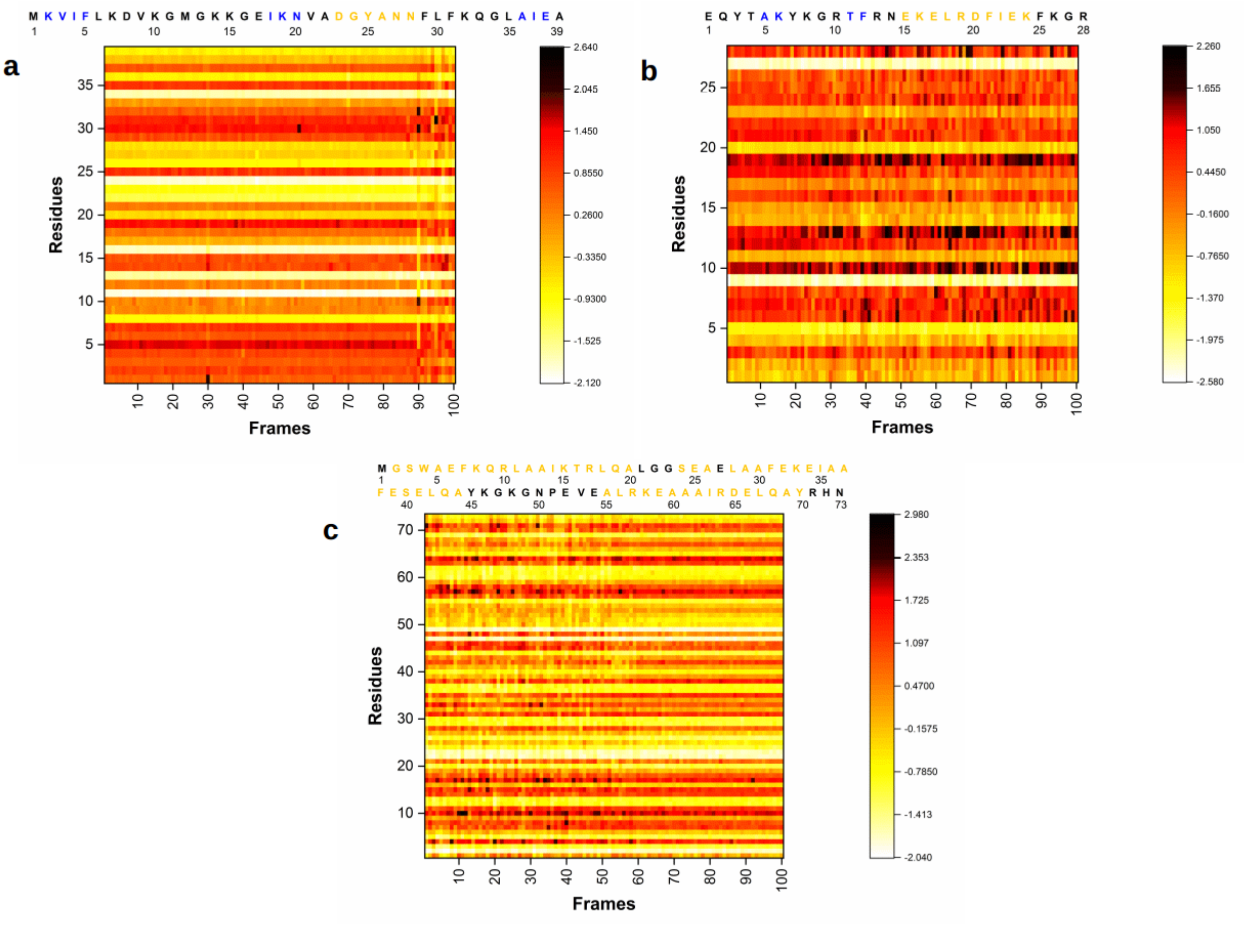

Figure S25: Local hardness heatmap for the proteins: (a) NTL9, (b) BBA, and (c) α3D obtained via the PM7 semiempirical method.

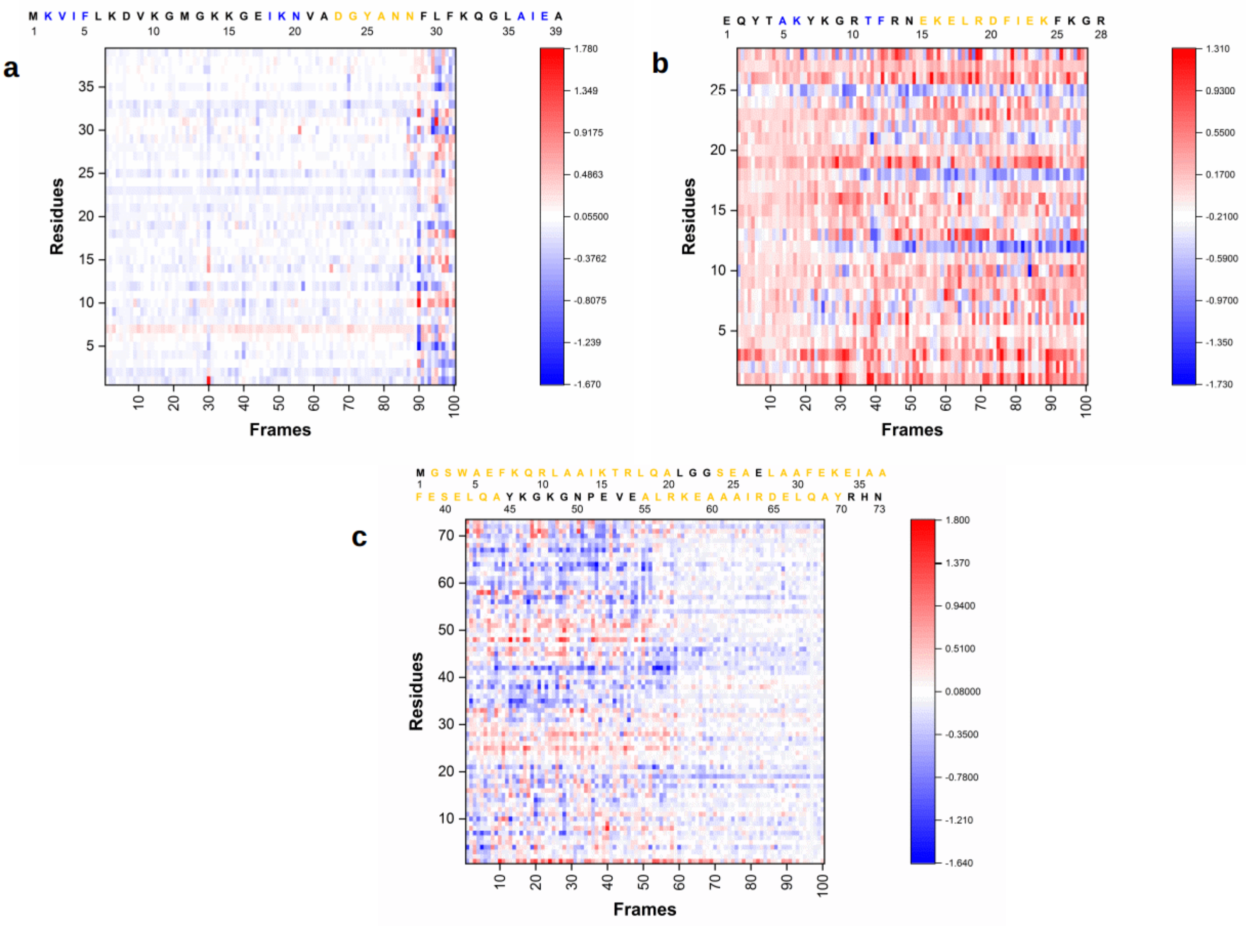

Figure S26: Heatmap of $\Delta\eta$ for the proteins: (a) NTL9, (b) BBA, and (c) α3D obtained via the PM7 semiempirical method.

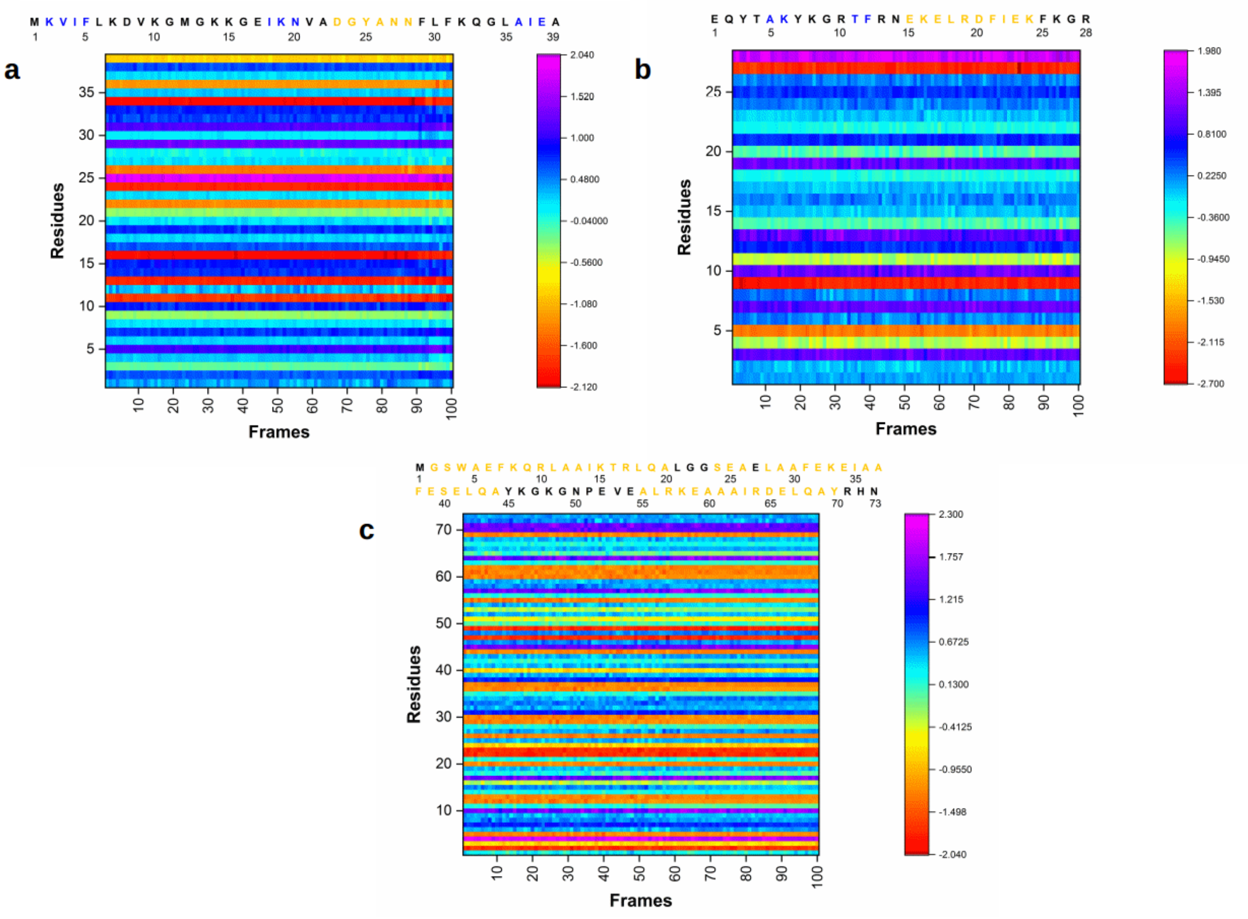

Figure S27: Local electron density heatmap for the proteins: (a) NTL9, (b) BBA, and (c) α3D obtained via the PM7 semiempirical method.

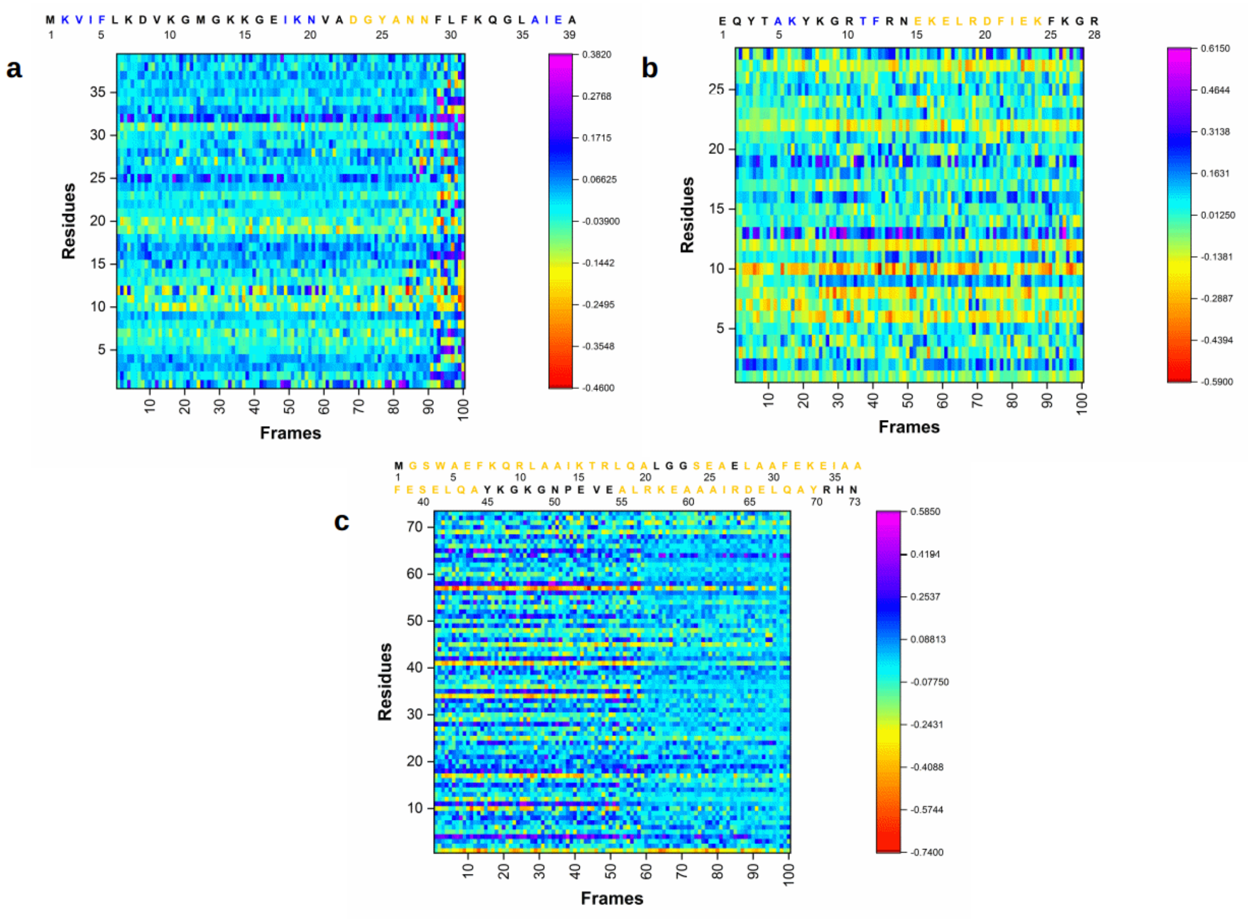

Figure S28: Variation in local electron density ($\Delta\rho$) heatmap for the proteins: (a) NTL9, (b) BBA, and (c) α3D obtained via the PM7 semiempirical method.

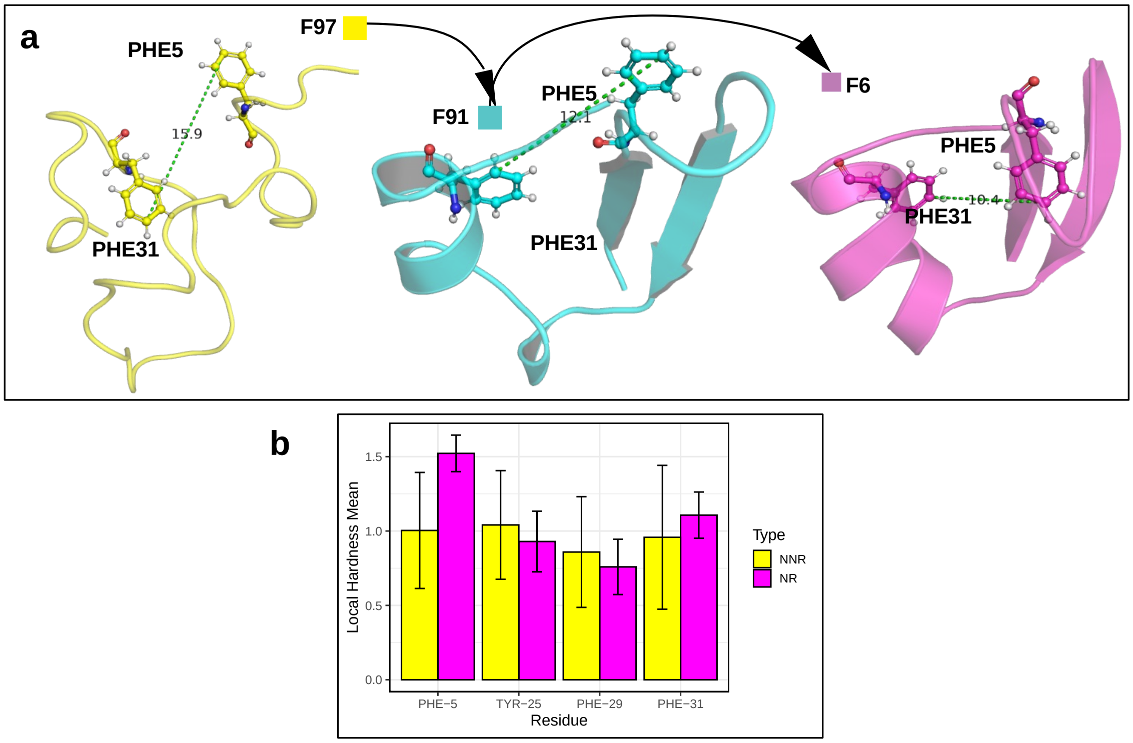

Figure S29: In (a): NTL9 protein conformations for frames 97, 91, and 6 with emphasis on residues Phe-5 and Phe-31. In (b): Average local hardness for group 2 residues of the NTL9 protein in the nonnative region (NNR) and native-like region (NR).

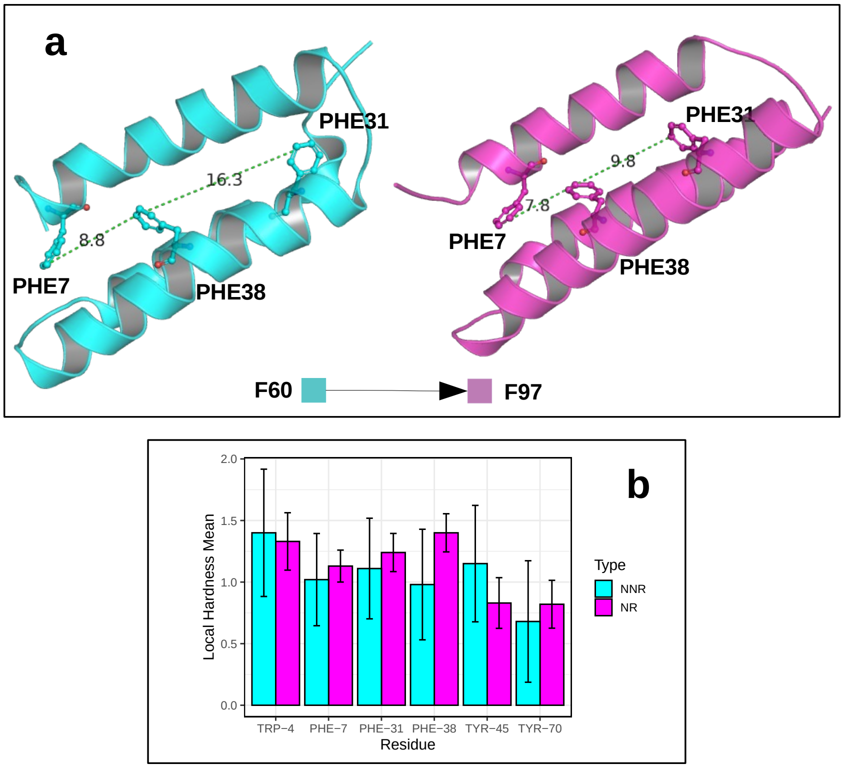

Figure S30: In (a): α3D protein conformations for frames 60 and 97 with emphasis on residues Phe-7, Phe-31, and Phe-38. In (b): Average local hardness for group 2 residues of the BBA protein in the nonnative region (NNR) and native-like region (NR).
